# MIZ-1 controls transcriptional programs required for BCR signaling, actin dynamics, and naïve B cell survival

**DOI:** 10.3389/fimmu.2026.1758550

**Published:** 2026-03-27

**Authors:** Julie Ross, Eva-Maria Piskor, Charles Joly-Beauparlant, Lucia Paola Gabrielli Tabarez, Elie El Ghorayeb, Arnaud Droit, Christian Kosan, Tarik Möröy

**Affiliations:** 1 Institut de recherches cliniques de Montréal (IRCM), Hematopoiesis and Cancer Unit, Montréal, QC, Canada; 2 Department of Medicine, Division of Clinical and Translational Research, McGill University, Montreal, QC, Canada; 3 Department of Biochemistry, Center for Molecular Biomedicine (CMB), Friedrich Schiller University Jena, Jena, Germany; 4 Bioinformatic platform, Faculty of Medicine, Université Laval, Québec, QC, Canada; 5 Department of Microbiology, Immunology, and Infectiology, Université de Montréal, Montréal, QC, Canada

**Keywords:** B cell activation, B cell receptor, Miz-1, signaling, survival

## Abstract

B cell receptor (BCR) signaling is required for the regulation of normal B lymphocyte development. However, many B cell lymphoma subtypes employ constitutive BCR signaling for transformation, survival, and growth. Here we report that mice expressing a POZ domain-deficient MIZ-1 (MIZ-1^ΔPOZ^), a well-known binding partner of the oncogene MYC, exhibit reduced surface receptor expression on naïve B cells and impaired BCR signaling. Transcriptomic and ChIP-seq analyses revealed that, in addition to autophagy related genes, MIZ-1 directly regulates genes involved in BCR signal transduction and actin cytoskeleton dynamics. Upon stimulation, MIZ-1^ΔPOZ^ B cells show defective receptor clustering and impaired BCR-induced signal transduction via key signaling molecules such as SYK, RAF1, AKT and ERK. We demonstrate that these perturbations are associated with altered calcium flux and mitochondrial respiration. These defects upon BCR crosslinking specifically lead to decreased survival of follicular but not marginal zone B cells and impaired the expansion of MYC-dependent B cell lymphoma. Altogether, our data suggest that MIZ-1 is as a central integrator of BCR signaling which is essential for maintaining B cell function and keeping a balance between cell death and survival.

## 1 Introduction

B cells are a central component of the adaptive immune system, responsible for the generation of antibodies that neutralize pathogens and facilitate their clearance (1). Both, B cell development and response to antigenic stimuli critically depend on signaling through the B cell receptor (BCR) (1, 2). Successful surface expression of a functional BCR marks the transition of immature B cells homing to peripheral secondary lymphoid organs, passing through transitional B stages 1-3 into two major subsets of naïve B cells with very different characteristics: follicular (FO) B cells and marginal zone (MZ) B cells (1, 3, 4). MZ B cells primarily perform T-cell independent responses to blood-borne antigens and pathogens in a rapid manner attributed to their broad repertoire of surface IgM receptors that can bind to conserved microbial antigens (5, 6), while FO B cells reside in the B cell follicles of the spleen, adjacent to T cell zones and are therefore primarily responsible for T cell-dependent antibody responses. After T-cell dependent activation of FO B cells, they form germinal centers where antibody affinity maturation and class switching occur, followed by the differentiation into plasma cells that secrete soluble antibodies with high affinity (7, 8). Although MZ and FO B cells carry out very different functions, the membrane-bound immunoglobulin is an essential regulator of their survival and differentiation. Here, weaker BCR signals combined with higher Notch2 signaling favor MZ development, while strong BCR signals favor differentiation of FO B cells (4, 9).

The B cell receptor is a non-covalent transmembrane complex of the diversified IgM for antigen recognition and a heterodimer of Igα (CD79A) and Igβ (CD79B) (10, 11). BCR ligation triggers the formation of `lipid rafts’ to increase intracellular signaling strength and signaling cascade that are crucial for B cell activation, proliferation, and differentiation (12). Transmembrane signal transmission is mediated by the phosphorylation of ITAMs (Immunoreceptor Tyrosine-based Activation Motifs), in the cytoplasmic tails on the Igα and Igβ subunits (9). The phosphorylation of Igα and Igβ ITAMs creates docking sites for recruitment of downstream effector kinases and allow Src family kinases (SFKs), like spleen tyrosine kinase (SYK) (11, 13, 14). Recruitment of SYK to Igα and Igβ initiates the activation of downstream cascades and signal transmission is facilitated by two signaling adaptor platform proteins, namely B cell linker protein (BLNK) and B cell adaptor for PI3K (BCAP), which feed into different downstream pathways (15, 16). The latter works in cooperation with the CD19 co-receptor and activation of the PI3K/AKT/mTOR1 axis by phosphorylation of its inhibitory subunit p85, enabling the phosphorylation of phosphatidylinositol-4,5-bisphosphate (PIP_2_) to phosphatidylinositol-3,4,5-trisphosphate (PIP_3_) to recruit AKT (16). Activation of AKT inhibits apoptosis and promotes mitochondrial remodeling to uphold the higher energy demand following activation (17–19). CD19/PI3K is not only important for proliferation and survival but is also a major component for survival of naïve peripheral B cells.

Stochastic triggering of the BCR pathway provides a continuous low-level antigen-independent BCR signal and mediates a `tonic’ signal, activating PI3K and its downstream effectors to ensure survival and inhibition of apoptosis in naïve B cells (20, 21). Equally important and central to maintain B cell function and responsiveness is the coordination of the endocytic machinery upon BCR ligation by actin polymerization, which enables membrane invagination and vesicle formation, to allow BCR internalization (22). Actin depolymerization facilitates BCR movement into endosomes for the processing and presentation of antigens or BCR degradation for signal termination to preserve cell responsiveness (23–25). This process represents a negative feedback loop following ligation, initiated by the SYK, BLNK and Phospholipase Cγ (PLCγ) signaling node, the other major downstream signaling cascade. Furthermore, accumulation of IP_3_ upon PLCγ2 activity provokes IP_3_R channel opening and Ca^2+^ release from endoplasmic reticulum (26). The increased Ca^2+^ pool contributes to mitochondrial remodeling to prepare for the higher energy demand following activation, as well as RAS, calcineurin and nuclear factor of activated T cells (NFAT) activation (27, 28).

Combined BLNK and BCAP initiated signaling cascades downstream of the BCR lead to changes in the metabolic and transcriptional profile that enable B cell activation and proliferation. One of the most significant factors to initiate proliferation and GC entry of activated B cells is the transcription factor MYC, which is very rapidly induced following BCR engagement (29). Upregulation of MYC in activated B cells enhances protein synthesis and glycolysis and allows activated B cells to expand, but at the same time, its explicitly stage-specific regulation in response to BCR signaling is critical for affinity-based selection (29–31). Upon activation, B cells specifically reprogram BCR signaling to attenuate BCR signaling via a negative feedback loop by rapid dephosphorylation of SYK with subsequent reduction of AKT phosphorylation and its downstream effectors to allow GC selection, affinity maturation and restrain MYC activity (32–34). After activation, the selection and differentiation of B cells depend on the cooperation of MYC and the MYC interacting Zinc-finger protein 1 (MIZ-1, ZBTB17), which cooperatively regulate transcriptional programs for selection and alter BCR signaling and promote survival of selected cells (35, 36).

Although being an essential component for B cell maturation, MYC is a well-known and potent driver of malignant transformation and B cell lymphomagenesis (37). In B cell lymphomas, *MYC* amplification requires the cooption of BCR hyperactivation leading to constitutive PI3K and SYK activation to control B cell lymphoma fitness and survival (38–40). This allows accumulation of MYC and leads to its invasion of active promotors and enhancers and thereby to a general transcriptional amplification (41). By this invasion, MYC alters the normal expression pattern of factors mediating physiological cellular functions like cell cycle, protein biosynthesis, metabolism and signal transduction ultimately leading to malignant transformation (42). Essentially, the known MIZ-1 functions overlap with critical steps involved in B cell activation that can lead to malignant transformation, positioning MIZ-1 as a critical component for B cell development (35, 36, 43, 44).

MIZ-1 is a ubiquitous transcription factor involved in lymphocyte differentiation (45–48). MYC/MIZ-1 complexes have been shown to repress MYC target genes and deletion of the MIZ-1 POZ domain impedes MYC driven tumorigenesis (47, 49–53). MIZ-1 is a transcriptional regulator, which forms homodimers via its POZ domain to be fully active and to regulate target genes through the recruitment of the histone acetyl transferase p300 (49, 54, 55) to its own cognate binding sites on chromatin, which are different from MYC binding sites (56). MIZ-1 has been described to regulate different genes in various context like cell cycle regulators (57–59), circadian clock regulators (60), *Ndrg2* (61), *Pex13* (62), *Pcdh10* (63), *Ace2*, *Kdm8* (64) and *Wif-1* (65). In B cells, we have previously shown that MIZ-1 regulates *Socs1*, *Bcl2* and *Rpl22* during B cell maturation (66, 67). In addition to its transcriptional regulation, cytoplasmic MIZ-1 has been reported to be regulated by association with microtubules and may activate gene transcription in response to changes in the cytoskeleton (68).

Here we report that the deletion of the POZ domain of MIZ-1 is impairing normal BCR signaling in naïve B cells affecting the activation and differentiation of FO B cells. MIZ-1 was found at promoters of several BCR associated genes and MIZ-1^△POZ^ expression correlated with a global deregulation of BCR-associated genes. We observed a decreased expression of IgM molecules on the surface of MIZ-1^△POZ^ B cells and, upon stimulation, an impaired ability to form BCR clusters. This was associated with decreased phosphorylation of kinases like RAF1, ERK and AKT and decreased Ca^2+^ flux, which are essential components of the BCR signaling cascade. Unexpectedly, genes involved in signaling pathways that regulate the dynamics of the actin cytoskeleton were also deregulated by MIZ-1^△POZ^ resulting in aberrant organisation of actin upon BCR stimulation. This general impairment of the BCR signaling pathway was associated with altered mitochondrial function and decreased survival.

## 2 Results

### 2.1 MIZ-1 transcriptionally regulates genes associated with BCR signaling

**Figure 1:**
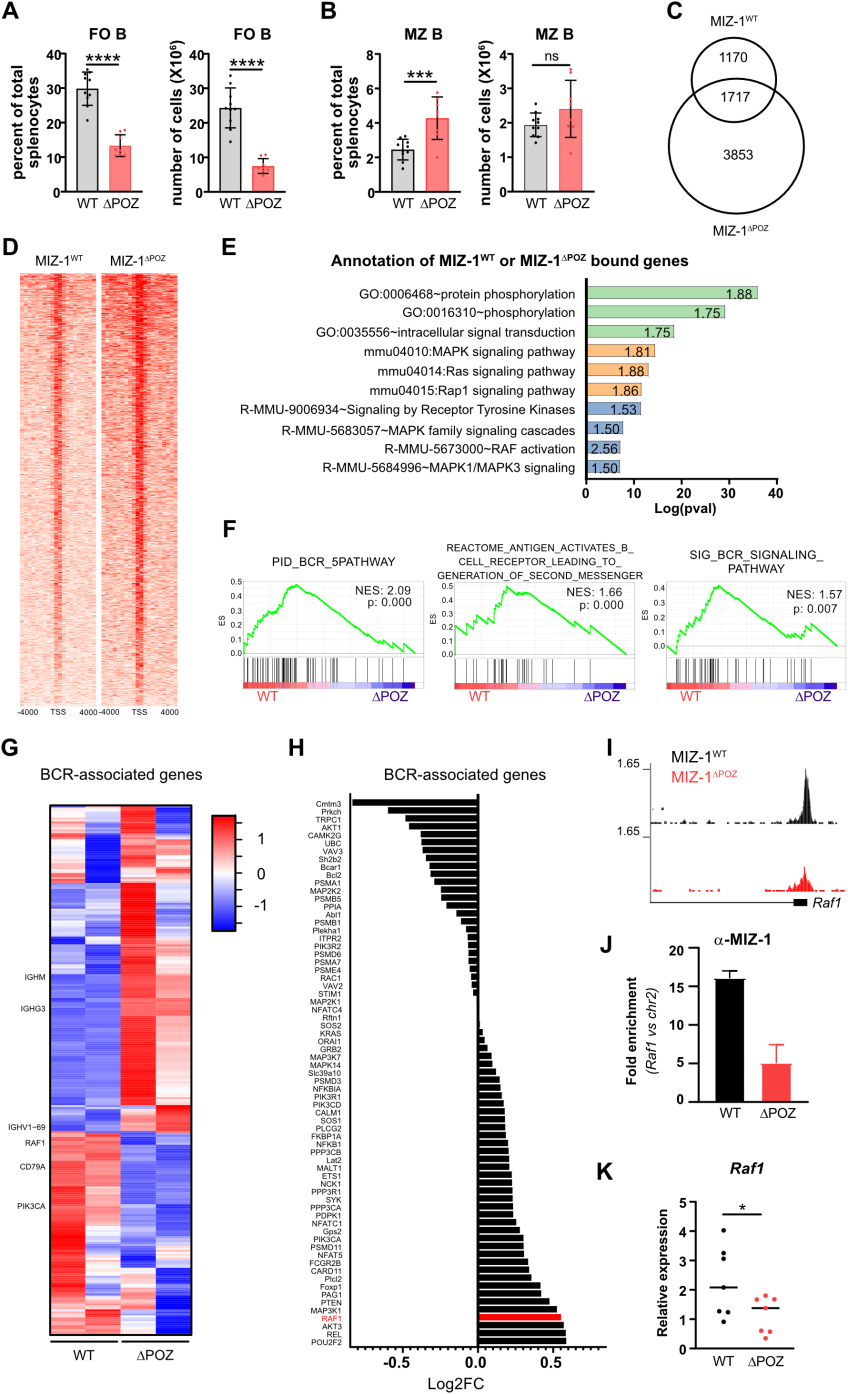
MIZ-1 transcriptionally regulates BCR signaling genes **(A, B)** FACS analysis of splenic B cell populations from MIZ-1^WT^ (WT) and MIZ-1^△POZ^ (△POZ) mice. Left panels show the percentage of indicated populations relative to total splenocytes and right panels show the number of cells of indicated populations in the spleen. FO B, follicular B cells (B220+ CD19+ CD21med CD23+ IgDhi); MZ B, marginal zone B cells (B220+ CD19+ CD21hi CD23- IgM+ IgDlo). Student’s unpaired t-test was used for statistical analysis (ns, not significant; ***p<0.001; ****p<0.0001) **(C–E)** ChIP-seq was performed on splenic B220+ cells from MIZ-1^WT^ and MIZ-1^△POZ^ mice using α-Miz-1 antibody. **(C)** Venn diagram showing the number of peak called in annotated transcriptional start sites (TSS) for MIZ-1^WT^ or MIZ-1^△POZ^ ChIP-seq. **(D)** Heatmap showing the recruitment of MIZ-1^WT^ and MIZ-1^△POZ^ to regions spanning +/- 5 kbp around annotated transcription start sites (TSS). Distance to TSS is indicated in base pairs. **(E)** Log(pvalue) of selected significantly enriched functions from DAVID Gene Annotation analysis (see **Supplementary Table 1**). Green: GO TERM Biological Process; Orange: KEGG Pathway; Blue: Reactome Pathway. Numbers in each bar indicates the Fold enrichment compared to background. **(F, G)** RNA-seq was performed on splenic B220+ cells from MIZ-1^WT^ and MIZ-1^△POZ^ mice. **(F)** Gene Set Enrichment Analysis (GSEA) for BCR related gene sets. NES: Normalized enrichment score (values indicate enrichment in MIZ-1^WT^); p, p value. **(G)** Heatmap showing RNA levels (z-score) of genes associated with BCR signaling (see **Supplementary Table 2**). **(H)** Log2FC values (MIZ-1^WT^ vs MIZ-1^△POZ^) extracted from RNA-seq analysis of genes associated with BCR signaling that are bound by MIZ-1^WT^ or MIZ-1^△POZ^. Positive and negative values indicate respectively a downregulation and an upregulation in MIZ-1^△POZ^. **(I)** Visualisation of MIZ-1 peaks at *Raf1* promoter from ChIP-seq in D. **(J)** Quantification of the recruitment of MIZ-1^WT^ or MIZ-1^△POZ^ by ChIP-qPCR to *Raf1* promoter in CD43- splenic B cells from MIZ-1^WT^ or MIZ-1^△POZ^ mice (n=3). Fold enrichment was calculated using 2^△△Ct^ method. Student’s unpaired t-test was used for statistical analysis (p = 0.09). **(K)** Relative quantification of *Raf1* mRNA levels in CD43- splenic B cells from MIZ-1^WT^ or MIZ-1^△POZ^ mice. Relative mRNA levels were calculated using 2^△Ct^ method. *Polr2a* was used as internal control. Student’s unpaired t-test was used for statistical analysis (*p< 0.05).

We had previously shown that in *Vav^Cre^ x Miz-1^floxPOZ^* mice, where the floxed POZ domain of *Miz-1* is deleted by Cre recombinase in all hematopoietic cells, the expression of a truncated MIZ-1 protein lacking the POZ domain results in a drastic decrease of B cell numbers in bone marrow and spleen (66). In *Mb1^Cre^ x Miz-1^floxPOZ^* mice, where the Cre recombinase is only active in B cells (**Supplementary Figures 1A, B**), we also observed a significant decrease of B cells compared to control mice (69). More specifically, in these animals, the FO B cell population was predominantly affected leading to a loss of cellularity in the spleen, while MZ B cells were increased in frequency (**Figures 1A, B**; **Supplementary Figures 1C, D**), while the overall architecture of a follicle was preserved in *MB1^Cre^ x Miz-1^floxPOZ^* mice (**Supplementary Figure 1E**).

To identify the mechanisms responsible for this phenotype, we performed a ChIP-seq analysis with WT and *MB1^Cre^ x Miz-1^floxPOZ^* B cells (hereafter called MIZ-1^WT^ and MIZ-1^△POZ^) using an anti-MIZ-1 antibody. The analysis of promoter regions from both groups revealed that 1170 were bound by MIZ-1^WT^ while 3853 were occupied by MIZ-1^△POZ^ and 1717 promotor regions showed overlapping binding of MIZ-1^WT^ and MIZ-1^△POZ^ (**Figures 1C, D**). More specifically, our ChIP seq experiment with B cells from WT mice identified 2887 annotated promoter regions occupied by MIZ-1. The experiment with B cells from *MB1^Cre^ x Miz-1^floxPOZ^* mice showed 5570 annotated promoter regions occupied by MIZ-1^ΔPOZ^. Comparing both experiments, we noted that 1717 annotated promoter regions can be occupied by MIZ-1 or MIZ-1^△POZ^. This indicated that the deletion of the POZ domain does not abrogate DNA binding of MIZ-1 but allows occupation of the MIZ-1^ΔPOZ^ protein at *de novo* sites. This was expected since the DNA binding zinc finger regions in the MIZ-1^△POZ^ protein are still present and intact and it has been reported before that a MIZ-1^△POZ^ protein can bind DNA (54).

Interrogation of the ChIP-seq data with the DAVID annotation tool revealed that many genes occupied by MIZ-1 or MIZ-1^△POZ^ were significantly enriched in categories belonging to intracellular signaling pathways initiated by RAS or receptor tyrosine kinases (**Figure 1E**; **Supplementary Table 1**). RNA-seq of splenic B cells of both groups showed that many genes showing an altered expression pattern in MIZ-1^△POZ^ B cells compared to B cells from MIZ-1^WT^ mice are associated with the BCR signaling pathway (**Figures 1F, G**; **Supplementary Table 2**). When we compared the lists of MIZ-1/MIZ-1^△POZ^ target genes from the ChIP-seq experiment with the RNA-seq data, we found that several differentially expressed genes are implicated in BCR signaling such as *Akt3, Rel*, and *Map3k1* (**Figure 1H**; **Supplementary Figure 1E**). The *Raf1* gene, which encodes an important mediator of BCR signaling required for activation of naïve B cells and differentiation (70, 71), showed strong binding by MIZ-1 at its promoter region and its expression was downregulated in MIZ-1^△POZ^ expressing cells. The reduced expression was accompanied by reduced recruitment of MIZ-1^△POZ^ to its promoter (**Figures 1I–K**). We also observed that the *Cd79a* gene, but not *Cd79b* gene, was downregulated in MIZ-1^△POZ^ cells although MIZ-1 did not bind to its promoter (**Figure 1G**; **Supplementary Figures 1F, G**). Since *Cd79a* (Igα; MB1) and *Cd79b* (Igβ; B29) encode for genes of the B cell receptor complex and the *Mb1^Cre^* allele is driven by the promoter of *Cd79a*, we investigated the level of expression of CD79A and CD79B proteins in MIZ-1^△POZ^ and MIZ-1^WT^ cells. We could confirm that both protein expression levels were not substantially altered by the *Mb1^Cre^* allele (72) and that CD79A and CD79B protein expression is not reduced in MIZ-1^△POZ^ cells (**Supplementary Figures 1H, I**).

The early transcriptional alterations following BCR mediated B cell activation occur within two hours of receptor ligation and lead to signal-specific changes of the transcriptomic landscape (73). We identified many of these BCR induced genes as MIZ-1 targets, which prompted us to assess their expression after BCR stimulation in MIZ-1^WT^ and MIZ-1^△POZ^ B cells (**Supplementary Table 3**; **Supplementary Figures 1G, H**). We found that *Kras* and *Psma7* were upregulated after BCR stimulation in MIZ-1^△POZ^ vs MIZ-1^WT^ B cells. Furthermore, we observed that anti-apoptotic *Bcl2* and *Pou2f2*, a key transcription factor for B cell development, were significantly downregulated when MIZ-1^△POZ^ is expressed in B cells. These results suggest that MIZ-1 is a key transcriptional regulator of BCR-associated genes at steady state and following B cell activation. In addition, our data demonstrate differential chromatin binding of MIZ-1^WT^ and that MIZ-1^ΔPOZ^, leading to distinct transcriptional changes in MIZ-1^△POZ^ B cells that perturb normal BCR signaling dynamics.

### 2.2 MIZ-1^△POZ^ expression impairs normal BCR signaling

**Figure 2:**
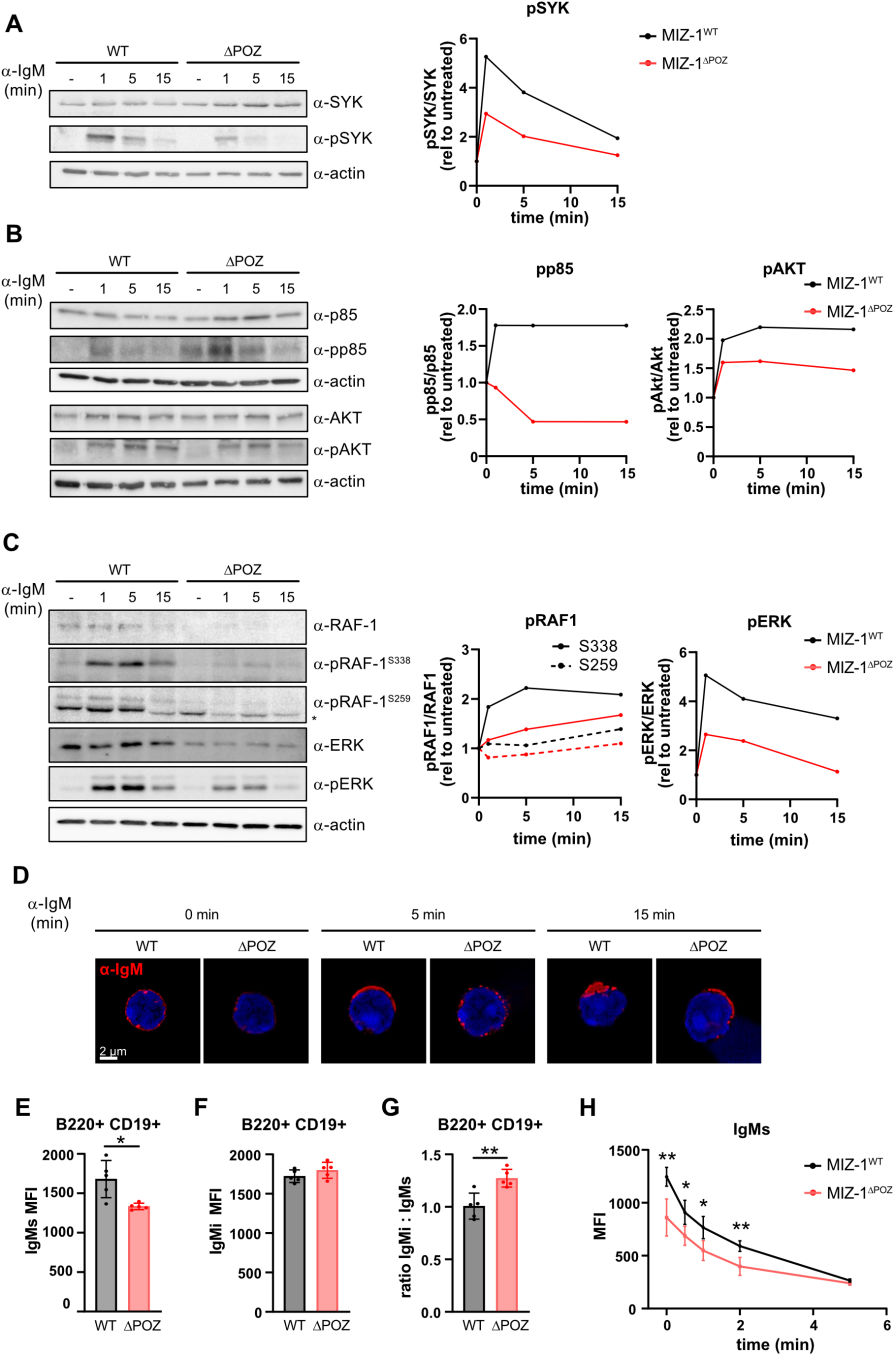
MIZ-1^△POZ^ impairs BCR signal transduction and clustering in B cells **(A–C)** left: Representative western blot showing expression of indicated proteins and the detection of their phosphorylated form in CD43- splenic cells from MIZ-1^WT^ and MIZ-1^△POZ^ mice stimulated or not with α-IgM (10μg/mL) for indicated time points. Actin was used as loading control. pSYK, SYK pY352; pp85, p85 pY458; pAKT, pS473; pRAF-1, RAF1 pS338 or pS259; pERK, ERK pT202/Y204. Right: quantification of left panels: fold increase of band intensity of phospho-protein relative to total protein and relative to untreated cells. (-): untreated; *: unspecific band; n=3. **(D)** Immunofluorescence showing localisation of BCR (IgM; red) in CD43- splenic cells from MIZ-1^WT^ and MIZ-1^△POZ^ mice before and after stimulation with α-IgM (10μg/mL) for indicated time points. The nucleus of the cells is stained with DAPI (blue). **(E, F)** Median fluorescence intensity (MFI) of IgM on resting B220+ CD19+ B cells from MIZ-1^WT^ and MIZ-1^△POZ^ spleens on the cell surface (IgMs) or internalized (IgMi) measured by FACS. **(G)** Ratio of IgMi vs IgMs from **(E)** and **(F)**. Student’s unpaired t-test was used for statistical analysis (*p<0.05, **p<0.01). **(H)** Internalisation assay showing median fluorescence intensity (MFI) of IgM on B cells (CD43-) from MIZ-1^WT^ and MIZ-1^△POZ^ mice at the cell surface (IgMs) after α-IgM stimulation for indicated time points measured by FACS. Ordinary two-way ANOVA with Sidak’s multiple comparisons test was used for statistical analysis (*p<0.05; **p<0.01).

Upon BCR stimulation, SYK kinase which acts upstream of different signaling cascades such as the PI3K/AKT pathway and the RAS/RAF/ERK pathway was rapidly phosphorylated (14). To measure the effect of MIZ-1^△POZ^ expression on BCR signaling effectors, we stimulated MIZ-1^WT^ and MIZ-1^△POZ^ B cells with F(ab’)2-α-IgM at different time points and assessed phosphorylation of SYK and downstream signaling effectors by Western Blot. The initial phosphorylation after stimulation of all tested effectors did not reach WT levels and stayed decreased over time in MIZ-1^△POZ^ B cells compared to MIZ-1^WT^ controls (**Figures 2A–C**). Additionally, the Y352 phosphorylation of the upstream kinase SYK was less efficient and SYK was more rapidly dephosphorylated in MIZ-1^△POZ^ B cells than in MIZ-1^WT^ B cells (**Figure 2A**). SYK dephosphorylation terminates the propagation of the BCR signal towards downstream kinases (74). Consequently, we observed decreased phosphorylation the p85 subunit of PI3K and AKT in MIZ-1^△POZ^ B cells compared to controls after BCR stimulation (**Figure 2B**) indicating a lack of proper BCR signal propagation. Similarly, we observed a decrease in p-RAF1 and p-ERK in stimulated MIZ-1^△POZ^ B cells (**Figure 2C**). These data suggest that aberrant transcriptional regulation of genes involved in BCR signaling in MIZ-1^△POZ^ B cells is associated with inefficient signal transduction after BCR stimulation. We can exclude that this is due to decreased expression of the BCR gene, as our RNA-seq data does not reveal a decrease of *Ighm* expression in MIZ-1^△POZ^ compared to MIZ-1^WT^ B cells. The differential signaling strength that we observe is consistent with the cellularity data (**Figures 1A, B**) that shows that FO B cells are less abundant in MIZ-1^△POZ^ mice and is in agreement with the established model that strong BCR signaling favors FO B cell fate, while weaker signals promote MZ differentiation (4). Thus, MIZ-1 appears to influence lineage decisions by modulating the strength and quality of BCR signaling.

After stimulation, ligated BCR molecules cluster and enter lipid rafts enriched with relevant downstream signaling molecules for signal amplification (75). Using immunofluorescence, we observed that BCRs on MIZ-1^△POZ^ B cells were unable to cluster and form lipid rafts as efficiently as WT controls (**Figure 2D**; **Supplementary Figures 2A–C**). The images also suggested that the surface presentation of BCRs is lower in MIZ-1^△POZ^ B cells. To confirm this observation, we measured the expression of IgM at the surface of naïve B cells (IgMs) and confirmed that BCRs are less abundant at the surface of resting MIZ-1^△POZ^ compared to MIZ-1^WT^ B cells (**Figure 2E**). Receptor internalization is part of a feedback mechanism, which is initiated by SYK dephosphorylation after ligand-induced activation and leads to the dissociation of SYK from the BCR complex allowing receptor internalization and signaling termination (74). We also measured intracellular IgM levels (IgMi) and observed an increased ratio of internalized BCR vs surface BCR in resting MIZ-1^△POZ^ compared to controls (**Figures 2F, G**). An internalization assay further showed that the kinetic of internalization of BCR after stimulation is significantly different in MIZ-1^△POZ^ B cells compared to MIZ-1^WT^ controls (**Figure 2H**), attributed to the higher level of intracellular IgM in MIZ-1^△POZ^ B cells compared to MIZ-1^WT^. In line with this, we find MIZ-1^△POZ^ B cells compared to MIZ-1^WT^ controls show reduced early endosome localization by co-localization with Rab5 shortly after stimulation (**Supplementary Figure 2B**), while co-localization with the lysosome marker LC3B is increased and confirms the increased intracellular IgM (**Supplementary Figure 2C**).

These results indicate that MIZ-1 is not only required for the transcriptional regulation of genes involved in BCR signaling, but also for signal amplification involving receptor clustering and BCR internalization, as both processes are aberrant in MIZ-1^△POZ^ B cells. Our data also suggests that the kinetic of the phosphorylation profile of signaling components are reminiscent of the negative feedback loop applied by germinal center B cells to attenuate BCR signaling to restrain MYC activity (32–34).

### 2.3 MIZ-1 is involved in the regulation of actin cytoskeleton and BCR localization

**Figure 3:**
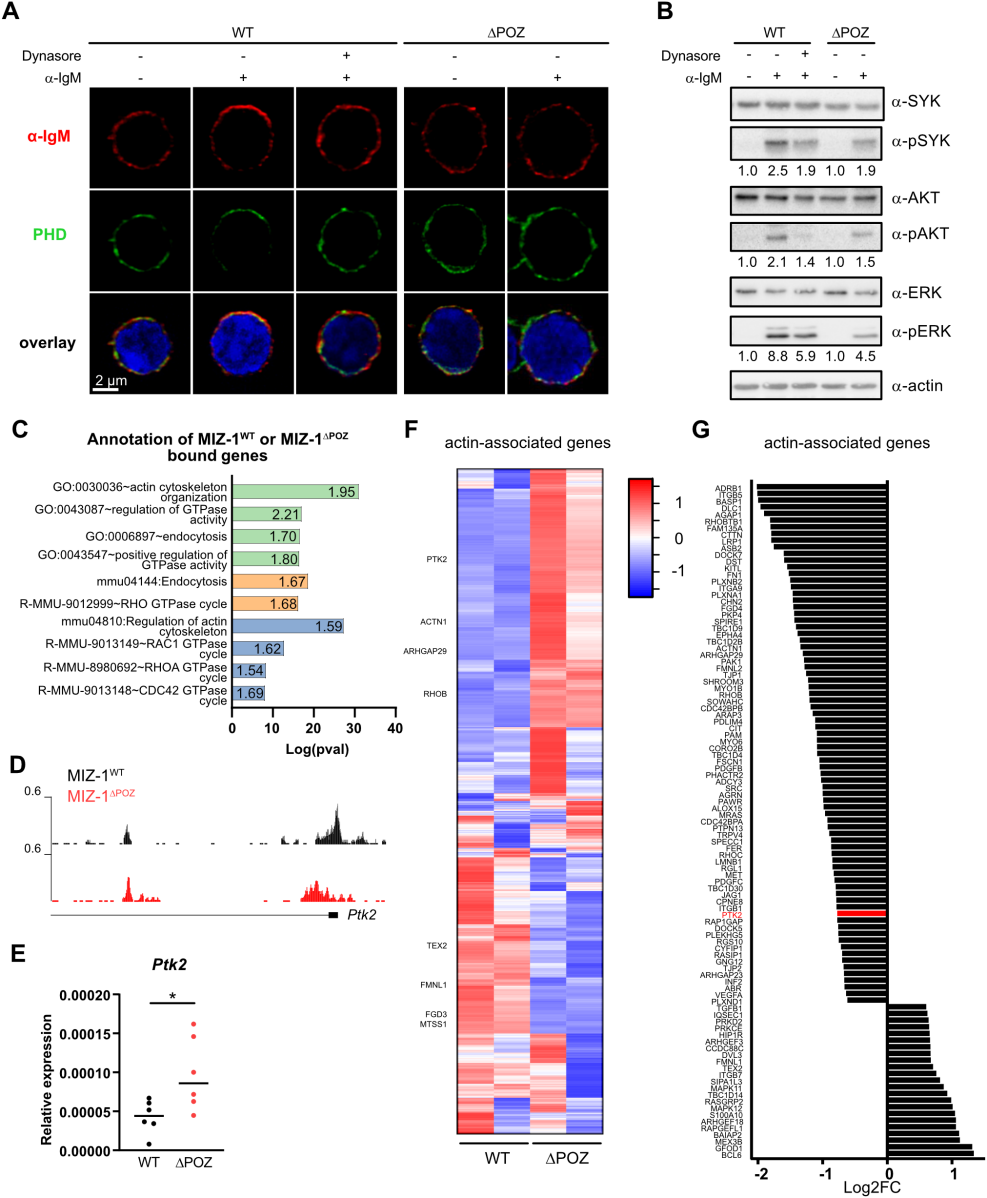
MIZ-1^△POZ^ interferes with BCR clustering upon stimulation. **(A)** Immunofluorescence showing localisation of BCR (IgM; red) or phalloidin (PHD; green) in CD43- splenic cells from MIZ-1^WT^ and MIZ-1^△POZ^ mice after stimulation with α-IgM (10μg/mL) for 1 minutes in presence (+) or absence (–) of dynasore. The nucleus of the cells is stained with DAPI (blue). **(B)** Western blot showing expression of indicated proteins and the detection of their phosphorylated form in CD43- splenic cells from MIZ-1^WT^ and MIZ-1^△POZ^ mice stimulated with α-IgM (10μg/mL) for 10 minutes in presence (+) or absence (–) of dynasore. Actin was used as loading control. pSYK, SYK pY352; pAKT, pS473; pERK, ERK pT202/Y204. **(C)** Log(p-value) of selected significantly enriched functions from DAVID Gene Anotation analysis of MIZ-1^WT^ and MIZ-1^△POZ^ ChIP-seq (see **Figure 1D** and **Supplementary Table 1**). Green, GO TERM Biological Process; Orange, KEGG Pathway; Blue, Reactome Pathway. Numbers in each bar indicate the Fold enrichment. **(D)** Visualisation of MIZ-1 peaks at *Ptk2* promoter from ChIP-seq in **Figure 1D**. **(E)** Relative quantification of *Ptk2* mRNA levels in CD43- splenic B cells from MIZ-1^WT^ or MIZ-1^△POZ^ mice. Relative mRNA levels were calculated using 2^△Ct^ method. *Rna18s1* was used as internal control. Unpaired t-test was used for statistical analysis (*: p<0.05). **(F)** Heatmap showing RNA levels (z-score) of genes associated with actin cytoskeleton regulation (see **Supplementary Table 2**). **(G)** Log2FC values (MIZ-1^WT^ vs MIZ-1^△POZ^) extracted from RNA-seq analysis of genes associated with actin cytoskeleton regulation that are bound by MIZ-1^WT^ or MIZ-1^△POZ^. Positive and negative values indicate respectively a downregulation and an upregulation in MIZ-1^△POZ^.

It was previously reported that, once internalized, BCRs continue to promote signaling from endosomes where RAF1 acts upstream of this signaling (76). We postulated that the aberrant signaling observed upon BCR stimulation in MIZ-1^△POZ^ B cells could be due to the negative feedback loop initiated by SYK dephosphorylation for BCR rewiring and endocytosis or due to the inefficient formation of BCR clusters. To test this, we used Dynasore, a dynamin inhibitor that blocks endocytosis, BCR cluster formation and destabilizes the actin cytoskeleton (77, 78) (**Figure 3A**; **Supplementary Figure 2B**). We observed that SYK, AKT and ERK phosphorylation was decreased upon BCR stimulation in normal B cells in the presence of Dynasore (**Figure 3B**). This was consistent with previous reports (76) and indicated that the altered BCR signal propagation observed in MIZ-1^△POZ^ B cells is similar to the signal observed in B cells that were treated with Dynasore, which impairs BCR molecule trafficking and actin cytoskeleton dynamics (**Figures 3A, B**; **Supplementary Figure 2B**). Stimulation of the BCR with soluble antigen induces actin reorganization that is required for BCR polarization (79). Furthermore, the dynamics and composition of lipid rafts, the signaling platforms for BCRs, are dynamically regulated by the actin cytoskeleton (80, 81). When we analyzed actin localization by immunofluorescence, we observed that actin organization after BCR stimulation is aberrant in MIZ-1^△POZ^ B cells and does not show a polarization with the BCR as compared to controls (**Figure 3A**; **Supplementary Figure 2B**). Interestingly, actin organization after BCR stimulation in MIZ-1^△POZ^ B cells was similar to MIZ-1^WT^ B cells, when BCR internalization was inhibited by treatment with Dynasore in MIZ-1^WT^ B cells (**Figure 3A**; **Supplementary Figure 2B**).

BCR clustering and endocytosis require a dynamic reorganization of the cytoskeleton (82, 83). MIZ-1 had previously been associated with tubulin and microtubule dynamics (68), which lead us to investigate whether MIZ-1 could regulate this process. An inspection of the list of promoters that are bound by MIZ-1 in B cells revealed an important number of genes associated with the actin cytoskeleton and RHO- or RAC GTPase pathways required for cell movement, shape and growth (**Figure 3C**; **Supplementary Table 1**). For example, *Ptk2* (focal adhesion kinase, FAK), a downstream effector of the BCR (84, 85), is upregulated following MIZ-1^△POZ^ binding to its promoter (**Figures 3D, E**). Many other actin cytoskeleton regulatory genes, such as *Spire1, Cdc42bpa, Arhgef18, Fmnl1* or *Tex2* were also deregulated in naïve MIZ-1^△POZ^ B cells (**Figures 3F, G**; **Supplementary Figure 2C**; **Supplementary Table 2**) or differentially expressed after BCR stimulation in B cells lacking a functional MIZ-1, versus controls such as *Itgav, Rapb1, Evl* and *Tnk2* (**Supplementary Figures 2D, E**; **Supplementary Table 3**).

A negative feedback loop between actin remodeling and BCR signaling in B cells controls actin dynamics, which initially promote activation and later contribute to downregulation of the signal (82). It involves actin-mediated BCR clustering and the recruitment of signaling molecules, followed by B-cell contraction, increasing the molecular density in clusters, ultimately leading to signal attenuation through favoring accessibility of interaction sites with inhibitory phosphatases in contracted clusters. Our data indicates that the transcriptional regulatory activity of MIZ-1^△POZ^ perturbs the expression of genes encoding proteins required for actin remodeling and thereby impede actin-dependent processes following BCR ligation and it is conceivable that MIZ-1^△POZ^ is directly responsible for aberrant actin organization seen in B cells that lack a functional MIZ-1. In addition, cytoplasmic MIZ-1 has been reported to activate gene transcription in response to changes in the cytoskeleton (68), which may further contribute to the observed phenotype.

### 2.4 MIZ-1^△POZ^ expression impairs molecular pathways downstream of BCR

**Figure 4:**
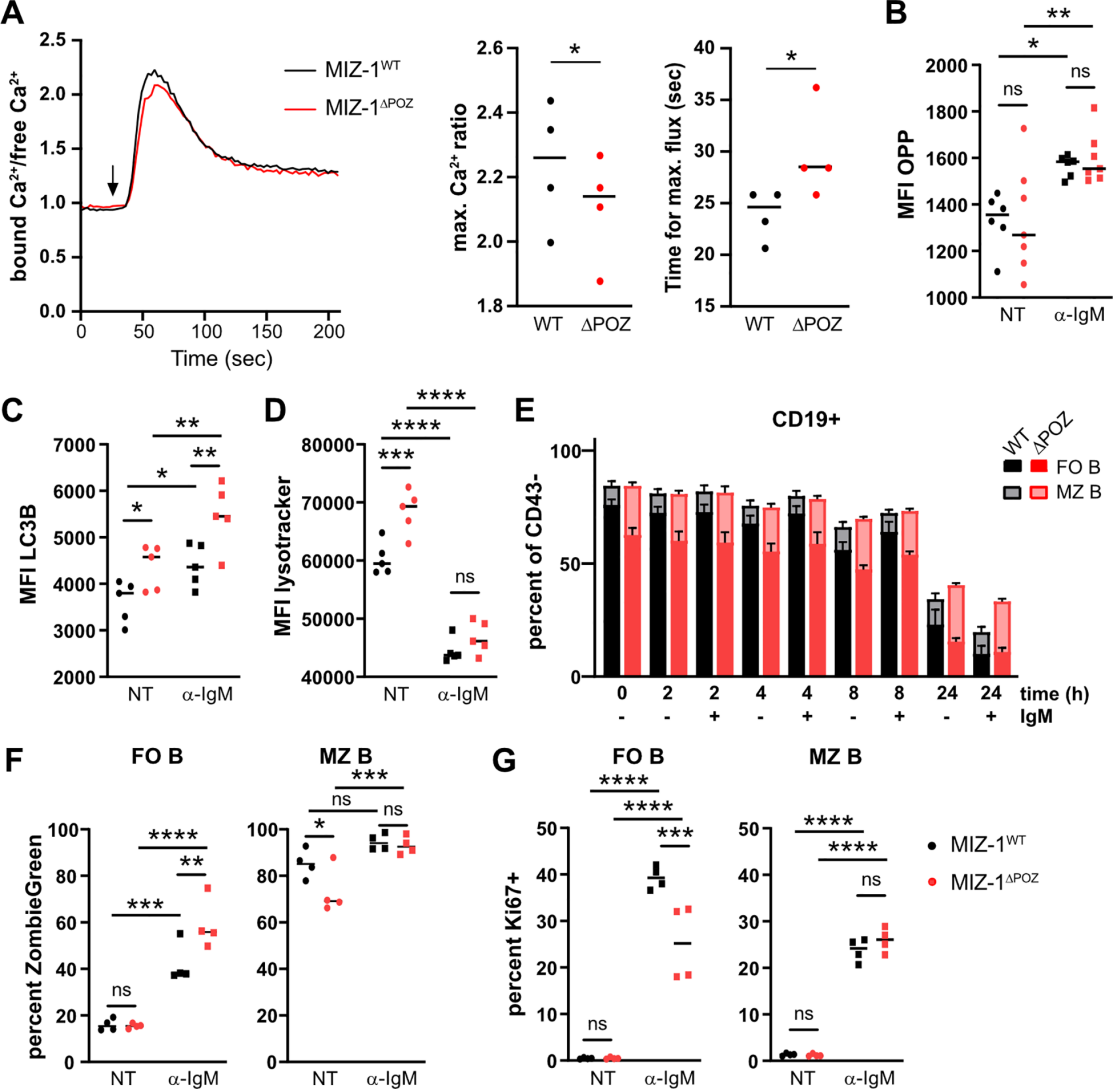
Impaired BCR signaling in naïve MIZ-1^△POZ^ B cells results in decreased Ca^2+^ flux, impaired autophagic flux and loss of follicular B cells. **(A)** Intracellular Ca^2+^ flux in CD43- splenic cells from MIZ-1^WT^ and MIZ-1^△POZ^ mice after stimulation with α-IgM (10μg/mL) was analyzed by the fluorescence emission shift of the calcium binding dye Indo-1 by FACS. Left panel: ratio of bound Ca^2+^ to free Ca^2+^ at indicated time points. The arrow indicates the time of stimulation. The graph represents the mean of 4 independent experiments. Middle panel: maximal ratio of bound Ca^2+^ to free Ca^2+^ was quantified at the timepoint of maximal flux. Right panel: time until maximal Ca^2+^ flux after stimulation. Student’s paired t-test was used for statistical analysis (*p<0.05) **(B–D)** CD43 depleted splenic cells from MIZ-1^WT^ and MIZ-1^△POZ^ mice were stimulated or not with α-IgM (10μg/mL) for 2h. FACS was used to measure OPP incorporation **(B)**, LC3B **(C)** or Lysotracker **(D)**. MFI, median fluorescence intensity; NT, Not treated. Ordinary two-way ANOVA with uncorrected Fisher’s LSD was used for statistical analysis (ns, not significant; *p<0.05; **p<0.01; ***p<0.001) **(E–G)** CD43 depleted splenic cells from MIZ-1^WT^ and MIZ-1^△POZ^ mice were stimulated or not with α-IgM (10μg/mL) for the indicated time points **(E)** or 48h **(F–G)**. Cells were stained for CD19, CD21 and CD23 **(E)** and then stained for Zombie Green and Ki67 **(F, G)** and percent of cells were measured by FACS. Ordinary two-way ANOVA with uncorrected Fisher’s LSD was used for statistical analysis (ns, not significant; **p<0.01; ***p<0.001; ****p<0.0001) for **(F–G)** No statistical test was performed for E but standard deviation are shown.

We next tested which cellular pathways could be affected by the altered BCR signaling pattern observed in MIZ-1^△POZ^ B cells. Among the events that occur very rapidly upon BCR stimulation and downstream of SYK and PI3K phosphorylation are the release of Ca^2+^ ions and reactive oxygen intermediates (ROIs) from the endoplasmic reticulum (86). A flow cytometric test revealed that the expression of MIZ-1^△POZ^ in B cells dampens normal calcium flux after BCR stimulation (**Figure 4A**). Other downstream events of PI3K signaling include translation and autophagy. Using an OPP incorporation assay, we could exclude that protein synthesis is affected in MIZ-1^△POZ^ B cells (**Figure 4B**).

BCR stimulation of WT B cells shows the accumulation of the autophagic marker LC3B and a decrease in lysosome mass indicating increased autophagic flux regarding autophagosome to lysosome fusion for degradation (**Figures 4C, D**). However, in MIZ-1^△POZ^ B cells, LC3B was increased at steady state (unstimulated), indicating impaired autophagic degradation (**Figure 4C**). Similarly, lysosome mass was increased in unstimulated MIZ-1^△POZ^ vs MIZ-1^WT^ B cells (**Figure 4D**), confirming the critical role of MIZ-1 in maintenance of efficient autophagy, consistent with previous reports (54). While the lysosome mass was comparable in MIZ-1^△POZ^ vs MIZ-1^WT^ B cells after BCR stimulation, LC3B levels increased to significantly higher levels (**Figures 4C, D**). We also confirmed that some genes associated with autophagy were aberrantly expressed in MIZ-1^△POZ^ vs MIZ-1^WT^ B cells (**Supplementary Figures 3A, B**). These results indicate that altered autophagic flux observed in MIZ-1^△POZ^ B cells is most likely due to a direct transcriptional effect of MIZ-1 on lysosome and autophagy related genes rather than to impaired BCR signaling.

We observed the accumulation of BCR molecules inside the cell, may at least in part, be related to the defect of BCR trafficking and impaired autophagic flux contributing to continuous receptor blunting. This decreases BCR signaling and eventually decreases B cell survival. Moreover, decreased autophagy will result in a reduction of available nutrients that the cell needs for proliferation. Therefore, we assessed survival and proliferation following BCR stimulation. BCR-stimulated naïve B cells undergo massive apoptosis within 24h (**Supplementary Figure 3C**). More specifically, FO B cells, which do not only rely on BCR stimulation to survive, rapidly decrease when cultured *in vitro* and are more apoptotic when MIZ-1^△POZ^ is expressed (**Figure 4E**; **Supplementary Figure 3D**). After 48h stimulation, we also observed a greater proportion of dead MIZ-1^△POZ^ FO B cells. Surviving MIZ-1^△POZ^ FO B cells were less proliferating than their WT counterpart (**Figures 4F, G**). No difference was observed between MIZ-1^WT^ and MIZ-1^△POZ^ MZ B cells, which likely reflects the metabolic differences between FO vs MZ B cells in response to BCR stimulation (87, 88).

### 2.5 Mitochondrial function is dysregulated in MIZ-1^△POZ^ B cells

**Figure 5:**
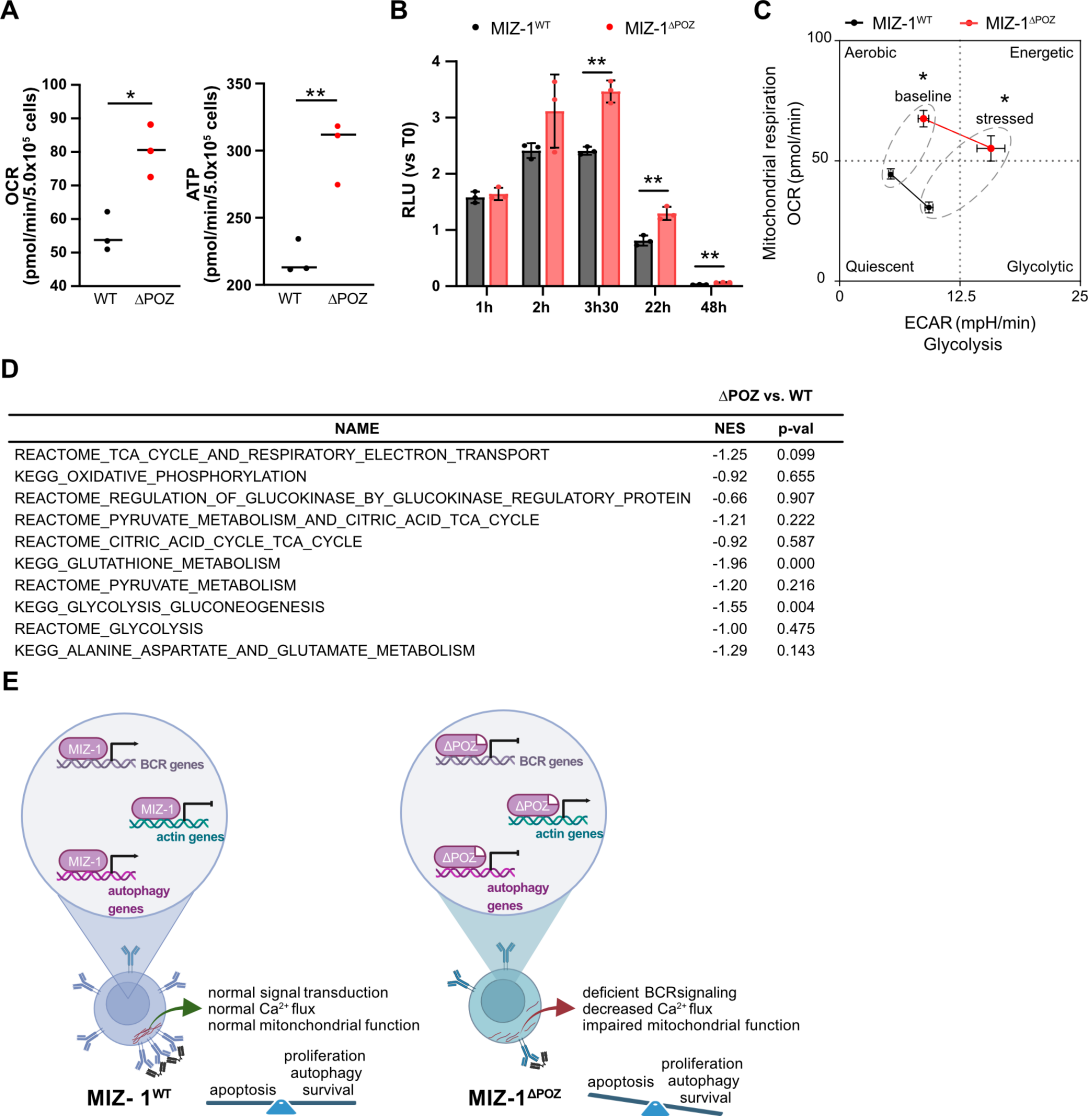
Dysfunctional mitochondrial activity in MIZ-1^△POZ^ B cells **(A, C)** Metabolic activity of CD43 depleted splenic cells from MIZ-1^WT^ and MIZ-1^△POZ^ mice analyzed by a Seahorse MitoStress test. **(A)** Quantification of the basal respiration and ATP production rates without additional stimulation. Student’s unpaired t-test was used for statistical analysis (*p<0.05). **(C)** Graphical representation of the metabolic flux characterized by oxygen consumptions rate (OCR) and extracellular acidification rate (ECAR). Baseline condition refers to initial metabolic activity. Stressed conditions represent metabolic activity after addition of carbonyl cyanide-p- trifluoromethoxyphenylhydrazon (FCCP) mimicking a physiological energy demand. Student’s unpaired t-test was used for statistical analysis (*p<0.05) **. (B)** ATP production was measured using CellTiterGlo on CD43 depleted splenic cells from MIZ-1^WT^ and MIZ-1^△POZ^ mice incubated in media for indicated time points. RLU, Relative luminescence; T0, time 0. Two-way ANOVA with Sidak’s multiple comparisons test was used for statistical analysis (**p<0.01). **(D)** Gene Set Enrichment Analysis (GSEA) of indicated functions from RNA-seq performed on splenic B220+ cells from MIZ-1^WT^ and MIZ-1^△POZ^ mice. NES, Normalized enrichment score (negative values indicate enrichment in MIZ-1^△POZ^ and positive values indicate enrichment in MIZ-1^WT^); p-val, p value. **(E)** In naïve B cells, MIZ-1^WT^ normally regulates genes involved in BCR pathway, actin pathway and autophagy pathway. Tight regulation of the expression of these genes is necessary for normal BCR signal transduction, actin cytoskeleton organisation and autophagic flux. These pathways favor normal activity of mitochondria and a balance between survival and cell death. When MIZ-1^△POZ^ is expressed, genes involved in BCR pathway, actin pathway and autophagy pathway are differentially expressed. This causes an inefficient BCR signal transduction, actin organisation and decreased autophagy that eventually results in mitochondrial dysfunction and increased cell death. Created with BioRender.com.

Downstream of the PI3K/AKT pathway, the mTOR complex is known to regulate mitochondrial biogenesis (89). Naïve B cells are normally in a relatively quiescent state characterized by moderate oxidative phosphorylation and low glycolysis (90). Upon BCR stimulation, these two pathways are activated to support higher energy demands and B cell mitochondria increase their oxygen consumption rate (OCR) to produce ATP (91). When we measured the OCR in resting B cells, we observed that MIZ-1^ΔPOZ^ B cells exhibited higher oxygen consumption and released greater amounts of ATP into the culture medium compared to controls, even in the absence of any stimulatory signal (**Figures 5A, B**). When the cells were stressed to mimic a higher energy demand, MIZ-1^△POZ^ B cells increased their glycolytic rate more significantly than MIZ-1^WT^ cells (**Figure 5C**). This metabolic reprogramming may be a compensatory response to impaired BCR signaling and autophagy, as B cells attempt to meet their energy demands despite reduced survival signals. The increased oxidative phosphorylation and glycolysis in MIZ-1^ΔPOZ^ B cells could therefore contribute to their reduced survival and increased apoptosis. Since we did not find a general deregulation of genes associated with mitochondria or glycolysis in MIZ-1^△POZ^ B cells (**Figure 5D**), these findings suggest an indirect link between the deregulation of BCR signaling in MIZ-1^△POZ^ B cells and their increased energy demand. It has been shown that BCR clustering and mitochondrial organization at the B cell synapse via the cytoskeleton are crucial for mitochondrial function and B cell survival (92). However, as deletion of the POZ domain leads to a perturbed actin cytoskeleton, MIZ-1^△POZ^ B cells have a decreased capability to reorganize mitochondria, which potentially contributes to an unfavorable metabolic outcome.

BCR signaling, mediating survival, and maintenance of metabolic homeostasis are key characteristics of MYC-driven lymphoma. It has been reported that Eμ-Myc mice, a mouse model for B cell lymphomagenesis (93, 94), show accumulation of splenic B cells in the pre-tumor phase. We observed that co-expression of MIZ-1^ΔPOZ^ in Eμ-Myc mice reversed this phenotype and correlated with increased B cell apoptosis. The residual splenic B cells in Eμ-Myc x MIZ-1^ΔPOZ^ mice displayed low MYC protein levels, suggesting a counterselection against high-MYC and a selective survival of low-MYC cells, if MIZ-1^ΔPOZ^ is expressed (**Supplementary Figure 4**). These findings indicated that MIZ-1 is essential for pre-lymphomatous Eμ-Myc B cell survival and support the therapeutic potential of targeting MIZ-1 in MYC-driven B cell malignancies.

## 3 Discussion

The present study describes the role of the zinc finger and BTB/POZ domain protein MIZ-1 in BCR signaling and metabolic pathways. Using mice that express a non-functional MIZ-1 protein due to deletion of its POZ domain (MIZ-1^△POZ^), we found that MIZ-1 deficiency leads to reduced B cell numbers, impaired BCR signaling, altered actin cytoskeleton dynamic resulting in aberrant metabolic activity, including increased mitochondrial respiration and glycolysis and decreased BCR expression at the cell surface. Upon BCR stimulation, we show that MIZ-1 is important to sustain calcium flux and autophagy as well as balancing apoptosis and proliferation. MIZ-1^ΔPOZ^ expression is associated with irregular clustering and internalization of the BCR, and reduced phosphorylation of key signaling molecules involving key pathways like the KRAS and PI3K pathway, highlighting MIZ-1’s critical role in maintaining BCR signaling and metabolic homeostasis.

MIZ-1^△POZ^ retains its zinc finger DNA binding domain but lacks the POZ domain that mediates homodimerization and coactivator recruitment. Our observation that loss of the POZ domain will eliminate a subset of WT MIZ 1 binding sites while also permitting non-canonical or POZ domain independent functions was therefore expected. Our downstream analyses were designed to incorporate both possibilities by analyzing the transcriptional consequences across all binding categories rather than relying solely on the overlapping peaks or peaks indicating only MIZ-1 DNA binding.

It is known that the consequences of impaired BCR signaling extend to B cell subset differentiation. In this context, it is conceivable that our finding of a marked reduction in follicular (FO) B cells and a relative increase in marginal zone (MZ) B cells in MIZ-1^ΔPOZ^ mice reflects this impairment of BCR signaling. This shift would be consistent with the established model in which strong BCR signaling favors FO B cell fate, while weaker signals promote MZ differentiation. Thus, MIZ-1 appears to influence lineage decisions by modulating the strength and quality of BCR signaling. Reduced expression of *Bcl2* and *Pou2f2* in MIZ-1^ΔPOZ^ FO B cells, which are essential for germinal center entry, suggests these cells are more prone to apoptosis and have a lower proliferation after BCR stimulation than MIZ-1^WT^ FO B cells.

As a transcription factor, MIZ-1 is known to regulate genes affecting cell cycle progression, differentiation, and apoptosis (46), while MYC is a well-established oncoprotein that drives cell proliferation, metabolism, and survival (95). In B cells, MYC overexpression is a hallmark of aggressive lymphomas, and its oncogenic function is tightly linked to BCR signaling, which provides survival and growth signals (96). The interplay between MIZ-1 and MYC is complex, as MIZ-1 can activate and repress MYC target genes, depending on the cellular context (66, 97). The data presented here indicate that MIZ-1 is crucial for maintaining proper BCR signaling, which is known to support MYC-driven oncogenesis, but the mechanism is independent of an interaction with MYC and has never been shown before. Our findings show that MIZ-1 occupies and deregulates a series of genes of the BCR signaling pathway, the actin cytoskeleton pathway, and that MIZ-1 deficiency is associated with reduced surface BCR expression, irregular BCR raft formation, and impaired phosphorylation of downstream kinases including SYK, p85, AKT, RAF1 and ERK support this notion. This finding is significant because BCR signaling has been shown to be essential for B cell survival, proliferation, and differentiation (33). Since in MYC-driven lymphomas, a functional BCR signaling is required to maintain the oncogenic activity of MYC (40, 98), our finding that a functional MIZ-1 is required to support BCR signaling offers an explanation of our previous reported finding that MYC driven lymphomagenesis is delayed or perturbed in MIZ-1^△POZ^ mice (49). Our finding that co-expression of MIZ-1^ΔPOZ^ in pre-lymphomatous Eμ-Myc mice correlated with increased B cell apoptosis supports this view. It suggests that a counterselection against high-MYC and a selective survival of low-MYC cells occurs when MIZ-1ΔPOZ is expressed. Furthermore, immunofluorescence data show less abundant BCRs on the surface of MIZ-1^△POZ^ B cells, impaired clustering, an aberrant organization and polymerization of actin molecules after BCR stimulation. Therefore, it is conceivable that these defects and the reduced amount of BCR molecules impede proper downstream kinase phosphorylation and signal amplification MIZ-1^△POZ^ B cells after stimulation. MYC upregulation following BCR ligation and signaling is critical to allow the expansion of activated B cells (29, 30). MYC is sequestered to the cytoskeleton to promote protein stability and disruption of the cytoskeletal dynamics promotes apoptosis (99). Similarly, the disrupted actin cytoskeletal dynamics in MIZ-1^△POZ^ B cells may impede proper MYC activity and therefore B cell proliferation.

The increased oxidative phosphorylation and glycolysis in MIZ-1^ΔPOZ^ B cells could contribute to their reduced survival and increased apoptosis, particularly in the context of MYC-driven lymphomas, where metabolic stress is already elevated. Since MIZ-1^ΔPOZ^ B cells exhibit increased mitochondrial respiration and glycolysis already in the absence of stimulation, the combined metabolic consequences are profound. The heightened metabolic state could reflect a compensatory response to impaired BCR signaling as an attempt by the cells to meet energy demands despite reduced survival signals. However, this metabolic reprogramming is not well adapted, since it is associated with increased apoptosis and impaired autophagic flux. This is most likely due to the function of MIZ-1 as a direct regulator of genes involved in lysosomal function and autophagy (54), suggesting that its role in metabolic homeostasis extends beyond BCR signaling.

Autophagy has been recognized as an essential component of B cell homeostasis preventing over stimulation as it drives the degradation of proteins, organelles, and pathogens that are associated with the B cell activation process (100). While autophagy is not the primary mechanism for the degradation of the BCR upon ligation, it has been shown to play a role in the regulation of BCR signaling by clearing damaged or aggregated receptor components and by acting as a quality control mechanism for other cellular components necessary for signal transduction (100). Moreover, autophagy is critical during early B cell activation and cell polarization in cooperation with the cytoskeletal reorganization, internalization and intracellular trafficking, which significantly affects signaling outcome (101, 102). In the context of our findings, it is thus conceivable that MIZ-1 indirectly participates in cell polarization, BCR trafficking and signal termination by controlling the expression of autophagy-related genes and cytoskeleton regulatory proteins.

Overall, we describe here the role of MIZ-1 in BCR signaling of naïve B cells by the transcriptional regulation of BCR-associated genes. A deregulation of this transcriptional program in MIZ-1 deficient B cells is associated with inefficient signal transduction upon BCR stimulation. We also found that MIZ-1 regulates several genes involved in the actin pathway and that a deregulation of this pathway in MIZ-1 deficient B cells was associated with altered BCR clustering and polarization upon BCR stimulation. As a consequence of this impaired BCR clustering and signaling, we show that Ca²^+^ flux is reduced following BCR ligation. After a strong BCR signal without a second stimulus, increased Ca^2+^ flux leads to mitochondrial dysfunction and cell death (103), but will also promote survival of a fraction of cells (104). In our experiments, we observed that MIZ-1 deficient B cells have greater mitochondrial dysfunction, increased apoptotic potential and decreased proliferative potential attributed to aberrant transcriptional programs leading to impaired BCR signaling, actin organization, autophagic flux and apoptosis in a cell-specific manner (**Figure 5E**). Interestingly, it was recently reported that MIZ-1 regulates expression of the *Tmbim4* gene in germinal center B cells to regulate Ca^2+^ release in response to selective, class-switched BCR activation (36). This finding further emphasizes the role of MIZ-1 in stage-specific regulation of BCR signaling in different B cell compartments to preserve their differentiation and survival potential.

## 4 Conclusion

Our study provides novel and hitherto unknown insight into the role of MIZ-1 in BCR signaling and B cell activation in naïve B cells. Importantly, the functional phenotypes we observe, reduced BCR signaling strength, impaired clustering, aberrant actin organization, defective autophagy, and decreased survival of follicular B cells, are all consistent with a loss of the functions of MIZ-1 that depend on its POZ domain, which includes the ability of the MIZ-1ΔPOZ protein to acquire *de novo* binding sites. Together with previous findings that showed a role of MIZ-1 in B cell development (66), B cell lymphomagenesis (49), B cell aging, and B cell immunocompetence (69), further establishes MIZ-1 as an essential factor for this part of the adaptive immune response. The data from this study have significant implications for B cell malignancies, particularly MYC-driven lymphomas, which depend on BCR signaling for MYC amplification and expression (105), since we had previously shown that MIZ-1^ΔPOZ^ impairs MYC-driven lymphomagenesis (49). The current study offers a potential mechanistic explanation for these observations. Although a direct causal relation has to be confirmed by additional experiments, our data suggests that by disrupting BCR signaling, MIZ-1 deficiency undermines important survival and proliferative signals that are required for MYC-driven malignant transformation. Targeting BCR signaling has been suggested as a promising therapeutic target for B cell lymphoma using potent kinase inhibitors (106). However, kinase inhibitor treatment harbors a major disadvantage, as primary and acquired resistance limits the efficacy for cancer treatment (107). We believe that our findings highlight the potential of targeting the MIZ-1/MYC axis by targeting MIZ-1 as a therapeutic strategy in B cell malignancies circumventing acquired kinase inhibitor resistances.

## 5 Materials and methods

### 5.1 Mice

*Miz-1^floxPOZ^* mice have been described previously (66). *Miz-1^floxPOZ^* mice were crossed with MB1-Cre animals (72) to obtain *MB1-Cre x Miz-1^floxPOZ^* mice where POZ domain of MIZ-1 is deleted in B cells (*Miz-1^△POZ^*). All mouse lines are backcrossed on a C57BL/6 background. Experiments were carried on animals of 40 to 70 days old. Animals were maintained in a SPF+ animal facility in the Institute de recherches cliniques de Montréal (IRCM). The IRCM Animal Care Committee has approved the animal protocols under which all animal experiments in this study have been performed (protocol 2016-08, 2020-07 and 2024-08), and all animal experimental procedures were performed in compliance with the guidelines of the Canadian Council of Animal Care (www.ccac.ca).

### 5.2 Mouse cell isolation and culture

Spleens were manually disrupted with glass slides in PBS and filtered to obtain single cell suspension. Red cells were removed using red blood cell lysing buffer (Sigma). When needed, cells were either positively selected using B220-biotin antibody (Biolegend) or negatively selected using CD43-biotin antibody (BD Pharmingen). Cells were incubated with biotin coupled antibody for 25 minutes, followed by incubation with MojoSort Streptavidin Nanobeads (Biolegend) for additional 25 minutes and purified from single cell suspension using the MojoSort magnet. For B220 positive selection, cells bound to beads were used for further experiments. For CD43 negative selection, cells bound to beads were discarded and untouched cells were used for further experiments.

For *in vitro* experiments, cells were cultured in B cell media (RPMI 1640, 5mM L-Glutamine, 10mM Hepes, 1mM Sodium Pyruvate, 1% MEM Non-essential Amino Acids, 1% Penicillin/Streptomycin, 10% FetalGro) for the time required for the specific assay. For Dynasore treatment and BCR stimulation, 1x10^6^ CD43 depleted splenocytes in a concentration of 5x10^6^/ml cells were seeded in 48-well plate and incubated with 100μM Dynasore for 1h at 37 °C. For BCR stimulation, cells were pre-incubated for 30 minutes at 37 °C and then stimulated with 10mg/mL F(ab’)2-Goat anti-Mouse IgM. For co-treatment, F(ab’)2-Goat anti-Mouse IgM was added to cells after Dynasore treatment. The stimulation was stopped by addition of cold PBS.

### 5.3 Flow cytometry

All flow cytometry data were collected on a BD LSR Fortessa (BD Bioscience) and analyzed with FlowJo software.

#### 5.3.1 Extracellular staining

Three million red cell lysed splenocytes were incubated in PBS for 15 minutes at 4 °C with the following antibodies for the analysis of B cells: B220 PerCP Cy5.5, CD19 APC Cy7, IgM BV421, IgD BV605, CD21 APC, CD23 PE, CD93 PE Cy7 (Biolegend and BD Pharmingen). Unless described differently in the Fig legend, Follicular (FO) B cell compartment was defined as CD19+ CD21low CD23+ and marginal zone (MZ) B cell compartment was defined as CD19+ CD21hi CD23-.

#### 5.3.2 Intracellular CD79 staining

Splenocytes were stained with CD19 BV421 (Bioloegend) as described above and incubated with Cyto-Fast Fix-Perm solution (Biolegend) for 20 minutes at room temperature and permeabilized 1X Cyto-Fast Perm Wash solution (Biolegend) according to manufacturer’s instructions. After permeabilization, cells were incubated for 20 minutes with CD79a-PE or CD79b-APC (Biolegend) at room temperature. Cells were washed with 1X Cyto-Fast Perm Wash and resuspended in cell staining buffer (Biolegend) for analysis.

#### 5.3.3 Intracellular IgM staining

Splenocytes were stained for extracellular markers B220 AF700, CD19 APC Cy7, CD43 biotin, IgM PE Cy7 (clone II/41; IgMs), IgD BV605, CD23 PE, CD21 APC as described above. Biotin coupled antibody was detected using Streptavidin BV421. Cells were also incubated with cell death marker 7AAD (Biolegend) to exclude dead cells. Cells were then incubated with IC Fixation buffer (eBioscience) for 30 minutes at room temperature and permeabilized with 1X permeabilization buffer. (eBioscience). For intracellular IgM staining, cells were incubated with IgM-FITC (clone II/41; IgMi) antibody (eBioscience) for 30 minutes at room temperature. The detection of intracellular IgM (FITC) was possible because the surface IgM was already saturated with IgM (PE Cy7). Cells were washed with 1X permeabilization buffer (eBioscience) and resuspended in cell staining buffer (Biolegend) for analysis. A ratio of IgMi/IgMs was calculated by dividing the MFI (FITC) of IgMi by MFI (Pe Cy7) of IgMs for each mouse.

#### 5.3.4 BCR internalisation assay

Three million CD43 depleted splenocytes were incubated with 1mg F(ab’)2-Goat anti-mouse IgM (eBioscience) or 1mg goat serum (Sigma) in 1X PBS for 20 minutes on ice. Cells were washed with cold 1X PBS and incubated at 37 °C with warm B cell media for various time points to favor BCR internalisation. Cold 1X PBS was used to stop internalisation and cells were incubated with 1 mg secondary antibody α-goat AF546 (Invitrogen) for 20 minutes on ice to detect IgM that is still detectable at the surface.

#### 5.3.5 Calcium flux assay

CD43 depleted splenocytes were incubated with 0.5 μM Indo-1 AM Calcium sensor dye and Pluronic (Invitrogen) as loading adjuvant in equivalent concentration in B cell media for 30 minutes at 37 °C.

#### 5.3.6 OPP incorporation assay

Cells were seeded in 24-well plate at a concentration of 2x10^6^ cell/mL and treated with α-IgM as described above for 2h at 37°C. After treatment, cells were transferred in a 5mL FACS tubes and pelleted by centrifugation. Cells were incubated for 30 minutes at 37°C in 20 μM Click-iT OPP working solution (Invitrogen). After washing with 1X PBS, cells were fixed in 3.7% Formaldehyde (in 1X PBS) for 15 minutes at room temperature, washed with 1X PBS and permeabilized with 0.5% Triton (in 1X PBS) for 15 minutes at room temperature. After washing with 1X PBS, click reaction was carried out in the reaction cocktail (2% copper protectant, 0.25% AF594 Azide, 1X Reaction buffer additive, 1X reaction buffer; Invitrogen) for 30 minutes at room temperature. Cells were washed with rinse buffer (Invitrogen) and stained with 1X HCS Nuclear Mask blue (Invitrogen) in 1X PBS for 30 min at room temperature.

#### 5.3.7 LC3B staining

Cells were seeded in 48-well plate at a concentration of 5x10^6^ cells/mL and treated with α-IgM as described above for 2h at 37°C. After treatment, cells were transferred in a 5mL FACS tubes and pelleted by centrifugation. Cells were fixed in 3.7% Formaldehyde (in 1X PBS) for 15 minutes at room temperature. After washing with 1X PBS, cells were permeabilized with 0.25% Triton (in 1X PBS) for 15 minutes at room temperature. Cells were washed and incubated with 0.25mg of LC3B antibody (Invitrogen) or 0.25mg of Rabbit IgG for 30 minutes at room temperature, washed with 1X PBS and incubated with 0.125mg a-rabbit AF647 (Life technologies) for 30 minutes at room temperature. After washing with 1X PBS, cells were incubated with 200ng/mL DAPI (Life technologies).

#### 5.3.8 LysoTracker staining

Cells were seeded in 48-well plate at a concentration of 5x10^6^ cells/mL and treated with α-IgM as described above for 2h at 37°C. After treatment, cells were transferred in a 5mL FACS tubes and pelleted by centrifugation. Cells were stained with 50 nM Lysotracker (Invitrogen) for 1h at 37°C.

#### 5.3.9 Annexin V staining

Cells were seeded in 48-well plate at a concentration of 5x10^6^ cells/mL and treated with α-IgM for various time points. After treatment, cells were transferred in a 5mL FACS tubes and pelleted by centrifugation. Cells were stained with CD19 APC Cy7, CD21 APC and CD23 PE for 12 minutes at 4°C. After washing with 1X PBS, cells were incubated with FITC Annexin V in in 1X Annexin V Binding Buffer for 15 minutes at room temperature.

#### 5.3.10 Ki67 staining

Cells were seeded in 48-well plate at a concentration of 5x10^6^ cells/mL and treated with α-IgM for various time points. After treatment, cells were transferred in a 5mL FACS tubes and pelleted by centrifugation. Cells were stained with 1mL of ZombieGreen in 100mL 1X PBS for 15 minutes at room temperature. CD19 APC Cy7, CD21 APC and CD23 PE antibodies were added to the mix without washing and incubated for additional 15 minutes at room temperature. After washing with Cell staining buffer (Biolegend) were fixed with Fixation/Permeabilization solution (1:3 concentrate in diluent; Invitrogen) for 30 minutes at room temperature. Cells were washed with 1X permeabilization buffer (Invitrogen) and stained with 1.5mL Ki67 BV421 in 100mL 1X permeabilization buffer for 30 minutes at room temperature.

### 5.4 Metabolic flux assay

Oxygen consumption rate (OCR) and extracellular acidification rate (ECAR) of CD43 depleted splenocytes were measured with the Seahorse XFe96 Extracellular flux analyzer according to manufacturer’s instructions (Agilent). Seahorse XF Sensor cartridges were hydrated in XF Sensor calibrant over night at 37 °C without CO_2_. XFe96 cell culture microplates were coated with 22.4 μg/ml Cell-Tak for 25 min at RT and wells were washed twice with sterile H_2_O immediately before use. CD43 depleted splenocytes were resuspended in freshly prepared, pre-warmed assay medium (1X Seahorse XF RPMI, 1 mM XF Pyruvate, 2mM XF Glutamine, 10mM XF Glucose) and seeded at 5x10^5^ cells in 180 μl per well. Plates were incubated for 1 h at 37 °C without CO_2_ to allow settling and attaching of the cells. Plates were immediately measured using the standard assay layout for general metabolic flux and timely injection of 1.5 µM Oligomycin, 0.5 µM FCCP and 0.5 µM Rotenone/antimycin A comprised in the kit. The metabolic phenotype was assessed by plotting OCR and ECAR values of basal, before injection of the compounds described above, and stressed cells, revealing the differential energetic states.

### 5.5 Chromatin immunoprecipitation and ChIP-sequencing

Chromatin immunoprecipitation was carried out on B220+ splenocytes using Miz-1 antibody (H-190, Santa Cruz). Briefly, 10 million cells were fixed with 1% Formaldehyde (in 1X PBS) for 10 minutes with agitation at room temperature. Fixation was blocked by the addition of 125mM glycine for 5 minutes with agitation at room temperature. Cells were pelleted by centrifugation at 4 °C and snap frozen until utilization. Fixed cells were lysed subsequently with cell lysis buffer (5mM pipes pH8, 85mM KCl, 0.5% NP-40, 1X Protease inhibitors cocktail 10mM PMSF) and nuclei lysis buffer (50mM Tris, 10mM EDTA, 1% SDS, 1X Protease inhibitors cocktail 10mM PMSF) for 30 minutes on ice each. Samples were sonicated with Covaris (Peak power: 140; Duty: 10; Cycle: 200; Time: 600s) and DNA fragment size (between 200 and 500bp) was verified on 1.5% agarose gel or with High sensitivity DNA kit and Bioanalyzer 2100 (Agilent). Sonicated material was precleared with 1:1 blocked Dynabeads (20μL protein A Dynabeads, 20μL protein G Dynabeads, Invitrogen), 0.5% Triton, 5.4mM Tris pH8, 160mM NaCl, 1mM EDTA, 62μM EGTA, 1.9mg/mL BSA, 1.6mg/mL tRNA; incubated for 2h with agitation at 4 °C) for 2h with agitation at 4 °C. Unbound material was then incubated with Miz-1 antibody (Santa Cruz for ChIP-seq and Novus for ChIP-qPCR) for 2h with agitation at 4 °C. Protein-chromatin complexes were collected using blocked Dynabeads overnight with agitation at 4 °C. Complexes bound beads were then washed sequentially with washing buffers W1 (0.5% NP40, 150 mM KCl, 10 mM Tris pH8, 1 mM EDTA), W2 (0.5% Triton, 0,1% DOC, 100 mM NaCl, 10 mM Tris pH8), W3A (0.5% Triton, 0.1% DOC, 400 mM NaCl, 10 mM Tris pH8), W3B (0.5% Triton, 0.1% DOC, 500 mM NaCl, 10 mM Tris pH8), twice W4 (0.5% NP40, 0.5% DOC, 250mM LiCl, 10 mM Tris pH8, 1 mM EDTA, twice B4 (10 mM Tris pH8, 1 mM EDTA). Complexes were then eluted from beads using 1X TPE buffer (0,3% SDS, 50mM Tris pH8, 10mM EDTA, 0,4M NaCl) for 4h at 65 °C. RNAse and proteinase K were added to the mix for 1h at 42 °C. DNA was extracted using QIAquick kit (Qiagen). DNA was then used to prepare libraries (KAPA Hyper Kit) for NGS sequencing (pair-end, 50 cycles) on HiSeq 2000/2500 System (Illumina) (n=1) or used as a template for qPCR (n=3, biological replicates). Reads were trimmed using Trimmomatic v0.36 (108) using default parameters. Trimmed sequences were aligned on the mm10 genome using bwa v0.7.17 (109). MACS2 v2.1.2 (110) and UCSC’s bedClip and bedGraphToBigWig (111) were used for the peak calling steps and to produce genomic tracks. Peaks were annotated using the ChIPseeker R package (112). Peaks were considered as targeting promoter regions when they were located less than 2000 nucleotides before a transcription start site or less than 1000 nucleotides after. Heatmap was generated with the ComplexHeatmap v1.12.0 R package (113). All R analyses were performed with R v3.3.2 (114). Peaks were visualized with Galaxy (115). The enrichment of biological functions was established using the DAVID tool (116, 117). A p value< 0.05, a fold enrichment > 1.5 and a FDR< 0.1 were considered significant.

Data were deposited in the GEO database (118)(accession: GSE313262).

### 5.6 RNA isolation and RNA sequencing

For RNA isolation, B220+ or CD43- splenocytes were lysed in TRI Reagent (Sigma) and isolated RNA was cleaned using the RNeasy Mini Kit (Qiagen).

### 5.7 RNA sequencing

A biological duplicate of sequencing libraries was prepared from RNA extracts. Ribosomal RNA was depleted (Ribo-Zero™ Magnetic Gold Kit, Epicentre) and library was prepared using KAPA Stranded RNA-Seq Kit with RiboErase. NGS sequencing (pair-end 50 cycles) was done on HiSeq 2000/2500 System (Illumina) (n=2 biological replicates). Reads were trimmed using Trimmomatic v0.36 (108) using default parameters and were then mapped to the mm10 genome using the TopHat2 software v2.0.10 (119). Reads were processed with Samtools70 followed by mapping to Ensembl transcripts using HTSeq71 (120). Normalisation (TPM) and differential expression was tested using the DESeq R package72 (R Core Team 2015, http://www.r-project.org/) (121). Log2FC of ±0.6 and pvalue > 0.05 was considered significant. The enrichment of biological functions was established using the GSEA tool (Broad Institute, v2.2.1). Heatmaps were generated using Heatmap2 tool on Galaxy (113, 115) using z-score normalization and hierarchical clustering. Multiple-testing correction was applied for all omics analyses using the default procedures implemented in the respective tools. Specifically, ChIP-seq peak calling with MACS2 relied on false discovery rate (FDR) control using Benjamini–Hochberg–adjusted q-values. Differential expression analysis in RNA-seq was performed with DESeq2, which applies Benjamini–Hochberg correction to control the FDR. Functional enrichment analyses were corrected for multiple testing using FDR-based approaches in DAVID and GSEA, as reported by each tool. Data were deposited in the GEO database (118) (accession: GSE313263).

### 5.8 cDNA preparation

RNA was quantified using Nanodrop (Thermo) and 1mg of RNA was treated with DNAse (Invitrogen) and submitted to reverse transcription reaction using Sscript II (Invitrogen).

### 5.9 Real-time PCR

DNA from ChIP or cDNA was used as a template for PCR reaction in presence of specific primer sets and SybrGreen Master Mix (AppliedBiosystem) using QuantStudio 5 real-time PCR system (15sec 95°C, 45sec 60°C for 40 cycles). Fold enrichment for ChIP experiment was calculated using 2-△△Ct method.


 ΔCt =Ct target gene−Ct reference gene

ΔΔCt =ΔCt ChIP−ΔCt input

Relative expression for qRT-PCR was calculated using 2-△Ct.


ΔCt =Ct target gene−Ct reference gene

### 5.10 Genotyping

Mouse tail tips were incubated overnight in digestion buffer buffer (20mM Tris-HCl pH 8.35, 50mM KCl, 0.5mM MgCl_2_, 0.01% gelatin, 0.05% Tween-20, 0.05% NP-40, 50mg/mL proteinase K in H_2_O) at 56 °C. 1mL of this mix was used as template for PCR (15sec 95°C, 30sec 58°C, 1min 72°C for 35 cycles) using specific primers. PCR products were ran on 2% agarose gel containing ethidium bromide and visualized with BioVision gel documentation system (Vilber Lourmat).

### 5.11 Protein extraction and western blot

Cells were seeded in 48-well plate at a concentration of 5x106 cells/mL and treated with α-IgM for various time points. Cells were collected in 1.5mL cold Eppendorf tubes containing 1mL of cold 1X PBS and pelleted by centrifugation. Cells were lysed in RIPA buffer (150 mM NaCl, 50 mM Tris-HCl pH 7.4, 1% NP-40, 0.5% DOC, 0.1% SDS) containing 1X protease inhibitor cocktail, 10 mM b-glycerophosphate and 2 mM sodium orthovanadate for 20 minutes on ice. After a 5 second pulse with the Sonicator, extracts were spun 10 minutes at 13000rpm at 4° and supernatant was collected for immunoblot analysis. Protein lysates were diluted in Laemmli buffer to a final concentration of 1X Laemmli, boiled and separated a 10% acrylamide gel in running buffer (25mM Tris, 190mM Glycine, 3.5mM SDS). Proteins were transferred on a nitrocellulose membrane in a transfer buffer (25mM Tris, 190mM Glycine, 20% methanol). Membranes were blocked with 5% non-fat dry milk in TBS-T (25mM Tris, 120mM NaCl, 2.5mM KCl, 0.1% Tween, pH7.5) for 1 hour at room temperature with agitation. Membranes were incubated with primary antibody (1:1000, except actin at 1:10000) in 5% BSA in TBS-T for 1h at room temperature or overnight at 4°C with agitation. Membranes were washed 3 times with 1X TBS-T and incubated with HRP coupled secondary antibody (1:10000) for 1 hour at room temperature in 1X TBS-T. Proteins were detected with ECL reagent (Thermo) on ChemiDoc Imaging System (Bio-Rad). For some experiments, membranes were re-incubated with a primary antibody that recognize a protein of a different size and detected as described above. Representative images of for different experiments (biological replicates, see Figure legend) are shown in the manuscript. Quantification was performed with ImageLab. Corrected volume intensity (corr vI) was calculated by removing mean background intensity (bI) from volume intensity (vI) of each band and corrected to the area (a).


$$corr\;vI=\frac{\left(vI-bI\right)}{a}$$

Corrected volume intensity of phosphoproteins (pprot) was divided by the corrected volume intensity of the total protein (prot) to obtain a pprot/prot ratio.


$$ratio=\frac{pprot}{prot}$$

A ratio fold increase was calculated relative to untreated sample for each mouse line.


$$fold\;increase=\frac{ratio\;treated}{ratio\;untreated}$$

### 5.12 ATP consumption

Cells were seeded at a concentration of 0.25x10^6^ cells/100mL in a white 96-well plate in B cell media. After various time points, 100uL of CellTiter Glo was added to all wells and incubated for 2 minutes on a microplate shaker and 10 minutes without shaking at room temperature. Luminescence was measured with Glomax (Promega).

### 5.13 Immunofluorescence staining and microscopy

CD43 depleted splenocytes were seeded and stimulated with IgM as described above for the indicated time points. Cells were adhered to coverslips at 500 x g for 5min using a Cytospin. Adhered cells were fixed with 4% PFA in PBS for 15min at RT and washed 3x with PBS. Cells were permeabilized and blocked with 5% normal goat serum (Sigma #G9023) in PBS with 0.3% Triton-100 for 1h at RT and incubated with 1 μg/ml α-IgM antibody (Invitrogen, #A21426) alone or in combination with Rab5 (1:200, Cell Signaling #3547T) or LC3B (1:200, Cell Signaling #3868P) in blocking solution overnight at 4 °C. Next day, slides washed 3x with PBS with 0.3% Triton-100. For Rab5 and LC3B visualization, samples were incubated with 1 μg/ml α-rabbit IgG-Alexa Fluor Plus™ 647 (Invitrogen, #A32733TR) in blocking solution for 6h at 4 °C and washed 3x with PBS with 0.3% Triton-100. Actin was visualized with ActinGreen488 ReadProbes™ (Invitrogen, #R37110) according to manufactures instructions. Nuclei were counterstained with DAPI and mounted with Prolong™ Diamond Antifade Mountant (Invitrogen, #P36965). Images were acquired with a confocal Leica STELLARIS STED DMi8 fluorescence microscope in Lighting mode for adaptive deconvolution using a PLAN APO 63x/1.4 oil objective (Leica, #11506350) and sequential line acquisition. Images were further analyzed with Leica Application Suite X (LAS X) Software. For immunohistochemical analysis, tissue specimens were collected at the time of autopsy and processed for cryo-conservation. Spleens were equilibrated in O.C.T. medium (Sakura) for 5 minutes on ice, transferred to fresh O.C.T medium, snap-frozen over liquid nitrogen and stored at -80 °C. For immunohistochemical analysis, 8 µm section were mounted on commercially available charged microscopy slides and air-dried for 10 min at RT. Slides were wrapped airtight with aluminum foil and stored at -20 °C until stained. Tissue was fixed with ice-cold acetone (10 min, RT) and washed with TBS-T (3x 5min, RT, rocking). Specimens were blocked with 2% BSA in TBS-T for 1h at 4 °C and incubated overnight with fluorescence-coupled antibodies, diluted 1:200 in 1%BSA/TBS-T in a humid chamber at 4 °C. Sections were washed 3x 5min with TBS-T and nuclein were counterstained with 1 µg/ml DAPI in PBS (7min, RT) in a humid chamber. Tissue staining was conserved with ProLong™ Diamond Antifade Mountant (Invitrogen, #P36965) and air-dried for 24h at RT prior to analysis. Microscopic pictures were acquired with LSM 880, AxioObserver and Plan-Apochromat 20x/0.80 M27 objective. Optimal laser settings were adjusted to a wildtype control. Pictures were processed with ZEN Microscopy and image software.

### 5.14 Statistical methods

Statistical analyses were carried out using GraphPad Prism. Test that were used are indicated in each figure legend and data points are representative for the number of biological replicates.

#### 5.14.1 Flow cytometry

For analyses where only non-treated samples from different mouse lines where compared, Student’s unpaired t-test was used for statistical analysis. Between 3 and 10 biological replicates where analysed. For calcium flux assays, we calculated and compared the mean flux per genotype and the within-day difference (Miz ^ΔPOZ^ to Control) using Student’s paired t test, pairing by day to control for batch effects. Calcium flux experiments are highly batch specific, as they are sensitive to subtle changes in e.g. Indo-1 loading or temperature. Therefore, we used a paired t-test, as one Miz ^ΔPOZ^ is naturally matched to a control measurement taken the same day under the same batch conditions (same prep, reagents, instrument, temperature, etc.). The pairing (by day) treats “day” as a blocking factor, and we therefore tested for the within-day Miz ^ΔPOZ^ to Control difference while removing day-to-day variability (batch effects). Data sets for co-localization from microscopy images were tested for normality distribution and if appropriate tested with Mann-Whitney U-test, indicated in the figure legends.

For analyses where non-treated samples were compared with treated samples from different mouse lines, ordinary two-way ANOVA with uncorrected Fisher’s LSD was used for statistical analysis. For analyses where non-treated samples were compared with multiple time point treated samples from different mouse lines, ordinary two-way ANOVA with Sidak’s multiple comparisons test was used for statistical analysis. Between 4 and 6 biological replicates were analysed in each independent experiment. A least three independent experiments were performed for each panel.

#### 5.14.2 Metabolic flux assay and ATP consumption

Student’s unpaired t-test was used for statistical analysis. 3 biological replicates where analysed.

#### 5.14.3 Quantitative Real-Time PCR

For analyses where only non-treated samples from different mouse lines where compared, Student’s unpaired t-test was used for statistical analysis. Between 3 and 10 biological replicates where analysed.

For analyses where non-treated samples were compared with treated samples from different mouse lines, repeated measure two-way ANOVA with uncorrected Fisher’s LSD was used for statistical analysis. Between 4 and 6 biological replicates were analysed in each independent experiment.

## Data Availability

Data were deposited in the GEO database (Edgar R NAR 2002) (accession of ChIPseq: GSE313262 and accession of RNAseq: GSE313263).

## Supplementary material

Supplementary Table 1 MIZ-1 peak annotation

Supplementary Table 2 Lists of genes used to build heatmaps

Supplementary Table 3 Differentially expressed genes after BCR stimulation in naïve B cells

Supplitalicentary Figure 1 MIZ-1 transcriptionally regulates BCR signaling genes. **(A)** Genotyping of *Miz-1* locus in CD43 depleted splenic cells from *Mb1-Cre*, *Miz-1^floxPOZ^* and *Mb1-Cre Miz-1^floxPOZ^* mice. *Miz-1^WT^*: *Miz-1* WT allele; *Miz-1^floxPOZ^*: *Miz-1* with floxed POZ domain allele; *Miz-1^ΔPOZ^*: *Miz-1* allele with deleted POZ domain. Position of the molecular weight marker is indicated on the left (bp). **(B)** Western blot showing MIZ-1 protein expression in *Mb1-Cre*, *Miz-1^floxPOZ^* and *Mb1-Cre Miz-1^floxPOZ^* mice. Actin was used as a loading control. **(C)** FACS analysis of splenic B cell populations from *Mb1-Cre*, *Miz-1^floxPOZ^* and *Mb1-Cre Miz-1^floxPOZ^* mice. Left panels show the percentage of B220+ CD19+ cells in the spleen and right panels show the number of B220+ CD19+ cells in the spleen. Unpaired t-test was used for statistical analysis (ns: not significant, ****: p<0.0001). **(D)** Graph showing the repartition of the different B cell populations in CD43 depleted splenic cells from *MIZ-1^WT^* and *MIZ-1^ΔPOZ^* mice. Cells were stained with B220, CD19, CD21, CD23, IgD, IgM and CD93 and analysed by FACS according to (Srivastava *et al*, 2005): T1: transitional 1 (CD19+ CD21low IgM_hi_ CD23^-^ CD93+); T2: transitional 2 (CD19+ CD21low IgM_hi_ CD23+ CD93+); MZP: marginal zone progenitors (CD19+ CD21_hi_ IgM_hi_ CD23+ B220+); MZ: marginal zone (CD19+ CD21_hi_ IgM_hi_ CD23low B220+); FO I: follicular I (CD19+ CD21+ IgMlow IgD_hi_); FO II: follicular II (CD19+ CD21+ IgM_hi_ IgD_hi_). **(E)** Immunofluorescence of spleen from non-immunized *MIZ-1^WT^* and *MIZ-1^ΔPOZ^* mice. Cryosections (8 µm) were stained with antibodies targeting IgD (red) to identify B cell follicles, CD21 (green) in order to evaluate anatomical features of the respective structures. DAPI (blue) was used for nuclear counterstain. Images shown are representative for duplicate immunofluorescent analysis for each genotype. Scale bar: 50 µm. **(F)** Visualisation of MIZ-1 peaks at indicated promoter from ChIP-seq in Fig 1D. **(G)** RNA was extracted from CD43 depleted splenic cells from *MIZ-1^WT^* and *MIZ-1^ΔPOZ^* mice. Relative quantification of mRNA levels of indicated genes measured by RT-qPCR using 2^ΔCt^ method. *Gapdh* was used as internal control. Student’s unpaired t-test was used for statistical analysis (ns: not significant, *: p<0.05). **(H-I)** FACS analysis of CD79A (H) or CD79B (I) protein levels on B cells from indicated lines (left panel) and MFI of CD79A or CD79B on B cells from 5 animals from indicated lines (right panel). Dark symbol: male and blue symbol: fitalicale. Student’s unpaired t-test was used for statistical analysis (ns: not significant, *: p<0.05). **(J)** Visualisation of MIZ-1 peaks at indicated promoter from ChIP-seq in Fig 1D. **(K)** CD43 depleted splenic cells from *MIZ-1^WT^* and *MIZ-1^ΔPOZ^* mice were stimulated (IgM) or not (NT) with α-IgM 10 µg/mL for 2 h. Relative quantification of mRNA levels of indicated genes measured by RT-qPCR using 2^ΔCt^ method. *Rna18s1* was used as internal control. Repeated measures two-way ANOVA with uncorrected Fisher’s LSD was used for statistical analysis (ns: not significant, *: p<0.05; **: p<0.01; ***: p<0.001).

Supplitalicentary Figure 2 MIZ-1^ΔPOZ^ interferes with BCR clustering upon stimulation. **(A)** Immunofluorescence showing localisation of BCR (IgM; red) and p-Raf1 (green) in CD43^-^ splenic cells from *MIZ-1^WT^* and *MIZ-1^ΔPOZ^* mice after stimulation with α-IgM 10 µg/mL for indicated time points. The nucleus of the cells is stained with DAPI (blue). (-): no treatment. **(B)** Maximum intensity projection of immunofluorescence z-stack acquisitions showing localisation of BCR (IgM; red) and Rab5 (green) in CD43^-^ splenic cells from *MIZ-1^WT^* and *MIZ-1^ΔPOZ^* mice after stimulation with α-IgM 10 µg/mL for 0, 5 or 15 minutes. The nucleus of the cells is stained with DAPI (blue). Cells in dashed squares are shown enlarged below for detailed visualization. Quantification indicates the IgM and Rab5 co-localization by IgM+Rab5+ positive puncta per cell (indicated with arrow heads, 0 min (n_WT_=237, n_ΔPOZ_=191), 5 min (n_WT_=239, n_ΔPOZ_=197), 15 min (n_WT_=187, n_ΔPOZ_=199)). Scale bar: 2 µm. Each point represents one cell. Mann-Whitney U-test (ns: not significant, **: p<0.01, ****: p<0.0001). **(C)** Maximum intensity projection of immunofluorescence z-stack acquisitions showing localisation of BCR (IgM; red) and Rab5 (green) in CD43^-^ splenic cells from *MIZ-1^WT^* and *MIZ-1^ΔPOZ^* mice after stimulation with α-IgM 10 µg/mL for 0, 5 or 15 minutes. The nucleus of the cells is stained with DAPI (blue). Cells in dashed squares are shown enlarged below for detailed visualization. Quantification indicates the IgM and Rab5 co-localization by IgM^+^LC3B^+^ positive puncta per cell (indicated with arrow heads, 0 min (n_WT_=228, n_ΔPOZ_=170), 5 min (n_WT_=175, n_ΔPOZ_=162), 15 min (n_WT_=221, n_ΔPOZ_=197)). Scale bar: 2 µm. Each point represents one cell. Mann-Whitney U-test (ns: not significant, ****: p<0.0001). **(D)** Immunofluorescence showing localisation of BCR (IgM; red) or phalloidin (PHD; green) in CD43^-^ splenic cells from *MIZ-1^WT^* and *MIZ-1^ΔPOZ^* mice after stimulation with α-IgM 10 µg/mL for 5 minutes in presence (+) or absence (-) of dynasore. The nucleus of the cells is stained with DAPI (blue). **(E-F)** Visualisation of MIZ-1 peaks at indicated promoter from ChIP-seq in Fig 1D. **(G)** CD43 depleted splenic cells from *MIZ-1^WT^* and *MIZ-1^ΔPOZ^* mice were stimulated or not with α-IgM 10 µg/mL for 2 h. Relative quantification of mRNA levels of indicated genes measured by RT-qPCR using 2^ΔCt^ method. *Rna18s1* was used as internal control. Repeated measures two-way ANOVA with uncorrected Fisher’s LSD was used for statistical analysis (ns: not significant, *: p<0.05, ***: p<0.001).

Supplementary Figure 3 Impaired BCR signaling in naïve MIZ-1^ΔPOZ^ B cells results in impaired autophagic flux and loss of follicular B cells. **(A)** Visualisation of *Miz-1* peaks at indicated promoter from ChIP-seq in Fig 1D. **(B)** Log_2_FC values (*MIZ-1^WT^* vs *MIZ-1^ΔPOZ^*) extracted from RNA-seq analysis of genes associated with autophagy that are bound by *MIZ-1^WT^* or *MIZ-1^ΔPOZ^*. Positive and negative values indicate respectively a downregulation and an upregulation in *MIZ-1^ΔPOZ^*. **(C)** CD43 depleted splenic cells from *MIZ-1^WT^* were stimulated (dashed line) or not (solid line) with α-IgM 10 µg/mL for indicated time points. Annexin V as well as CD19 was measured by FACS. No statistical analysis was performed but standard deviation are shown. **(D)** CD43 depleted splenic cells from *MIZ-1^WT^* and *MIZ-1^ΔPOZ^* mice were stimulated with α-IgM 10 µg/mL for 24 h. Cells were stained for CD19, CD21, CD23 and Annexin V and percent of cells were measured by FACS. Student’s unpaired t-test was used for statistical analysis (****: p<0.0001).

Supplementary Figure 4 MIZ-1^ΔPOZ^ is associated with apoptosis of MYC-overexpressing B cells. **(A-B)** FACS analysis of splenic B cell populations from *MIZ-1^WT^* (WT), *MIZ-1^ΔPOZ^* (ΔPOZ), *Eμ-Myc* (MYC) and *Eμ-Myc MIZ-1^ΔPOZ^* (MYC ΔPOZ) mice. **(A)** percentage of CD19^+^ population relative to total splenocytes **(B)** percentage Annexin V^+^ cell from the CD19^+^ population. Ordinary one-way ANOVA with Tukey’s multiple comparisons test was used for statistical analysis (ns: not significant, *: p<0.05; **: p<0.01; ***: p<0.001; ****: p<0.0001). **(C)** Western blot showing MYC protein expression in B220^+^ splenocytes from *MIZ-1^WT^* (WT), *MIZ-1^ΔPOZ^* (ΔPOZ), *Eμ-Myc* (MYC) or *Eμ-Myc MIZ-1^ΔPOZ^* (MYC ΔPOZ) mice. Lamin was used as a loading control.
