# Supplementary figures and images for: MIZ-1 controls transcriptional programs required for BCR signaling, actin dynamics, and naïve B cell survival
Source: Front Immunol. 2026 Mar 27;17:1758550. doi: 10.3389/fimmu.2026.1758550 (PMC13067367; doi:10.3389/fimmu.2026.1758550)

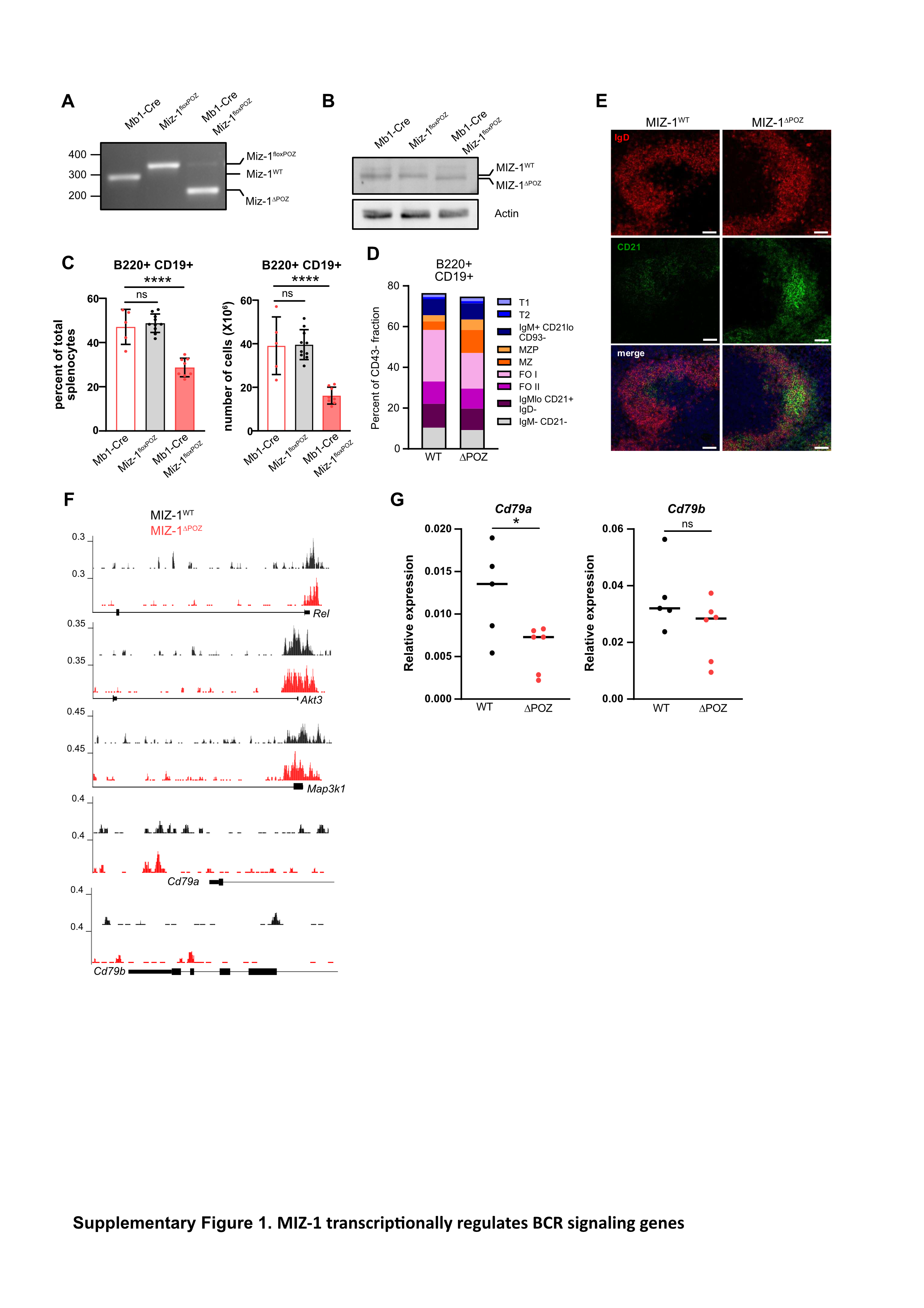

Supplement: Supplitalicentary Figure 1 — MIZ-1 transcriptionally regulates BCR signaling genes. (A) Genotyping of Miz-1 locus in CD43 depleted splenic cells from Mb1-Cre, Miz-1floxPOZ and Mb1-Cre Miz-1floxPOZ mice. Miz-1WT : Miz-1 WT allele; Miz-1floxPOZ : Miz-1 with floxed POZ domain allele; Miz-1ΔPOZ : Miz-1 allele with deleted POZ domain. Position of the molecular weight marker is indicated on the left (bp). (B) Western blot showing MIZ-1 protein expression in Mb1-Cre, Miz-1floxPOZ and Mb1-Cre Miz-1floxPOZ mice. Actin was used as a loading control. (C) FACS analysis of splenic B cell populations from Mb1-Cre, Miz-1floxPOZ and Mb1-Cre Miz-1floxPOZ mice. Left panels show the percentage of B220+ CD19+ cells in the spleen and right panels show the number of B220+ CD19+ cells in the spleen. Unpaired t-test was used for statistical analysis (ns: not significant, ****: p<0.0001). (D) Graph showing the repartition of the different B cell populations in CD43 depleted splenic cells from MIZ-1WT and MIZ-1ΔPOZ mice. Cells were stained with B220, CD19, CD21, CD23, IgD, IgM and CD93 and analysed by FACS according to (Srivastava et al, 2005): T1: transitional 1 (CD19+ CD21low IgMhi CD23- CD93+); T2: transitional 2 (CD19+ CD21low IgMhi CD23+ CD93+); MZP: marginal zone progenitors (CD19+ CD21hi IgMhi CD23+ B220+); MZ: marginal zone (CD19+ CD21hi IgMhi CD23low B220+); FO I: follicular I (CD19+ CD21+ IgMlow IgDhi); FO II: follicular II (CD19+ CD21+ IgMhi IgDhi). (E) Immunofluorescence of spleen from non-immunized MIZ-1WT and MIZ-1ΔPOZ mice. Cryosections (8 µm) were stained with antibodies targeting IgD (red) to identify B cell follicles, CD21 (green) in order to evaluate anatomical features of the respective structures. DAPI (blue) was used for nuclear counterstain. Images shown are representative for duplicate immunofluorescent analysis for each genotype. Scale bar: 50 µm. (F) Visualisation of MIZ-1 peaks at indicated promoter from ChIP-seq in Fig 1D. (G) RNA was extracted from CD43 depleted splenic cells from MIZ-1WT an [file Image1.tiff]

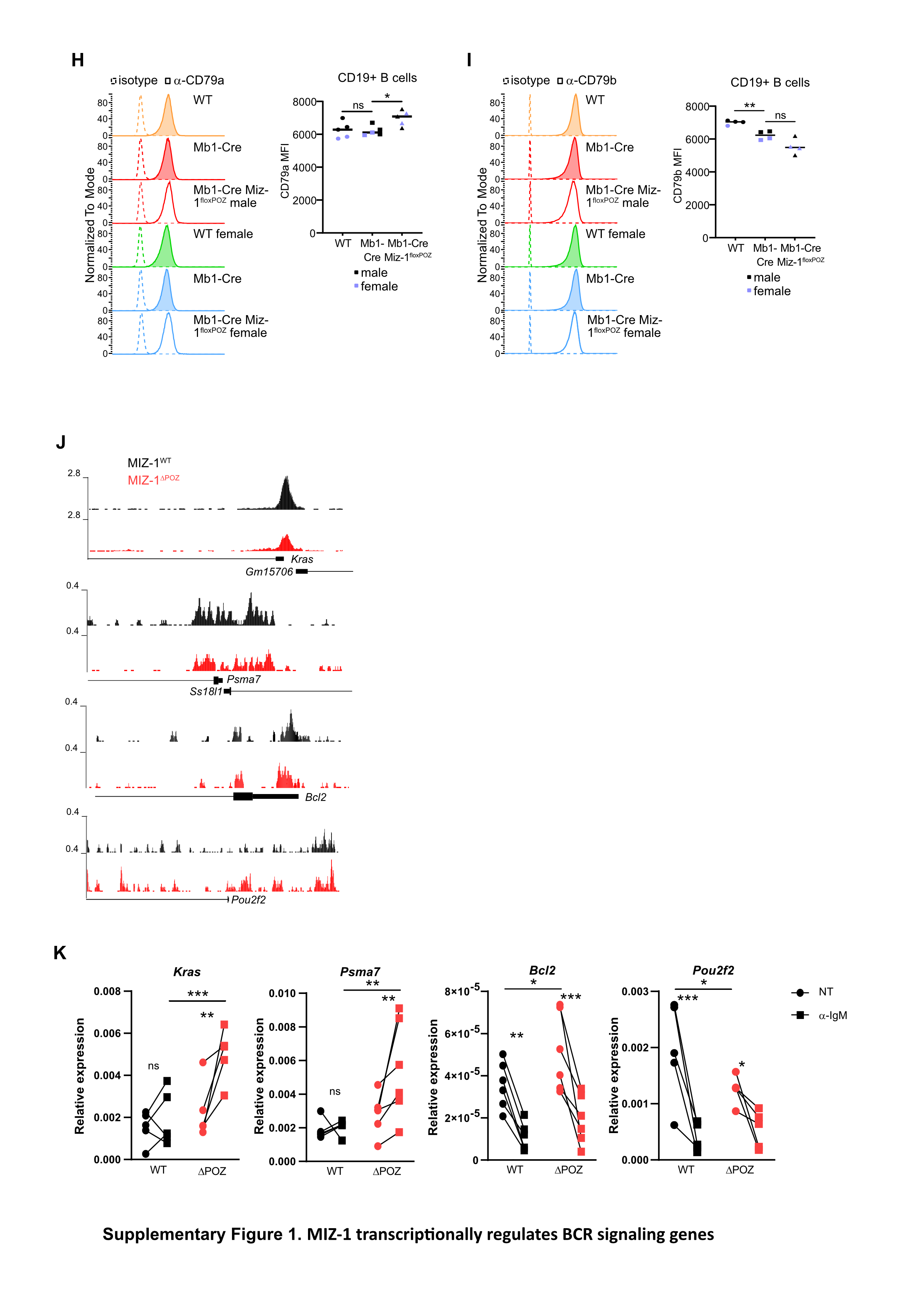

Supplement: Supplitalicentary Figure 2 — MIZ-1ΔPOZ interferes with BCR clustering upon stimulation. (A) Immunofluorescence showing localisation of BCR (IgM; red) and p-Raf1 (green) in CD43- splenic cells from MIZ-1WT and MIZ-1ΔPOZ mice after stimulation with α-IgM 10 µg/mL for indicated time points. The nucleus of the cells is stained with DAPI (blue). (-): no treatment. (B) Maximum intensity projection of immunofluorescence z-stack acquisitions showing localisation of BCR (IgM; red) and Rab5 (green) in CD43- splenic cells from MIZ-1WT and MIZ-1ΔPOZ mice after stimulation with α-IgM 10 µg/mL for 0, 5 or 15 minutes. The nucleus of the cells is stained with DAPI (blue). Cells in dashed squares are shown enlarged below for detailed visualization. Quantification indicates the IgM and Rab5 co-localization by IgM+Rab5+ positive puncta per cell (indicated with arrow heads, 0 min (nWT=237, nΔPOZ=191), 5 min (nWT=239, nΔPOZ=197), 15 min (nWT=187, nΔPOZ=199)). Scale bar: 2 µm. Each point represents one cell. Mann-Whitney U-test (ns: not significant, **: p<0.01, ****: p<0.0001). (C) Maximum intensity projection of immunofluorescence z-stack acquisitions showing localisation of BCR (IgM; red) and Rab5 (green) in CD43- splenic cells from MIZ-1WT and MIZ-1ΔPOZ mice after stimulation with α-IgM 10 µg/mL for 0, 5 or 15 minutes. The nucleus of the cells is stained with DAPI (blue). Cells in dashed squares are shown enlarged below for detailed visualization. Quantification indicates the IgM and Rab5 co-localization by IgM+LC3B+ positive puncta per cell (indicated with arrow heads, 0 min (nWT=228, nΔPOZ=170), 5 min (nWT=175, nΔPOZ=162), 15 min (nWT=221, nΔPOZ=197)). Scale bar: 2 µm. Each point represents one cell. Mann-Whitney U-test (ns: not significant, ****: p<0.0001). (D) Immunofluorescence showing localisation of BCR (IgM; red) or phalloidin (PHD; green) in CD43- splenic cells from MIZ-1WT and MIZ-1ΔPOZ mice after stimulation with α-IgM 10 µg/mL for 5 minutes in presence (+) or absence (-) of dynasore. The nucleus of th [file Image2.tiff]

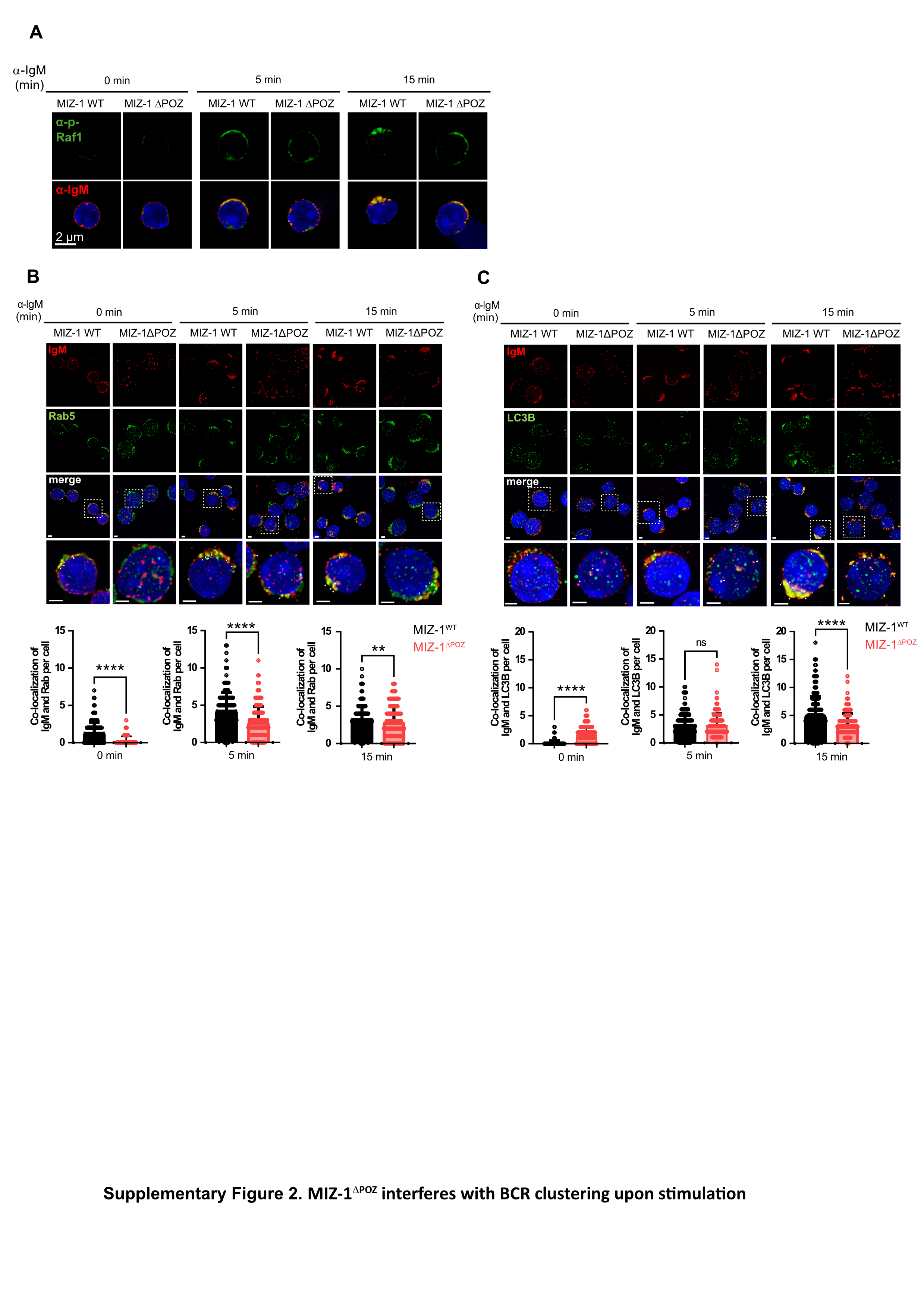

Supplement: Supplementary Figure 3 — Impaired BCR signaling in naïve MIZ-1ΔPOZ B cells results in impaired autophagic flux and loss of follicular B cells. (A) Visualisation of Miz-1 peaks at indicated promoter from ChIP-seq in Fig 1D. (B) Log2FC values (MIZ-1WT vs MIZ-1ΔPOZ ) extracted from RNA-seq analysis of genes associated with autophagy that are bound by MIZ-1WT or MIZ-1ΔPOZ . Positive and negative values indicate respectively a downregulation and an upregulation in MIZ-1ΔPOZ . (C) CD43 depleted splenic cells from MIZ-1WT were stimulated (dashed line) or not (solid line) with α-IgM 10 µg/mL for indicated time points. Annexin V as well as CD19 was measured by FACS. No statistical analysis was performed but standard deviation are shown. (D) CD43 depleted splenic cells from MIZ-1WT and MIZ-1ΔPOZ mice were stimulated with α-IgM 10 µg/mL for 24 h. Cells were stained for CD19, CD21, CD23 and Annexin V and percent of cells were measured by FACS. Student’s unpaired t-test was used for statistical analysis (****: p<0.0001). [file Image3.tiff]

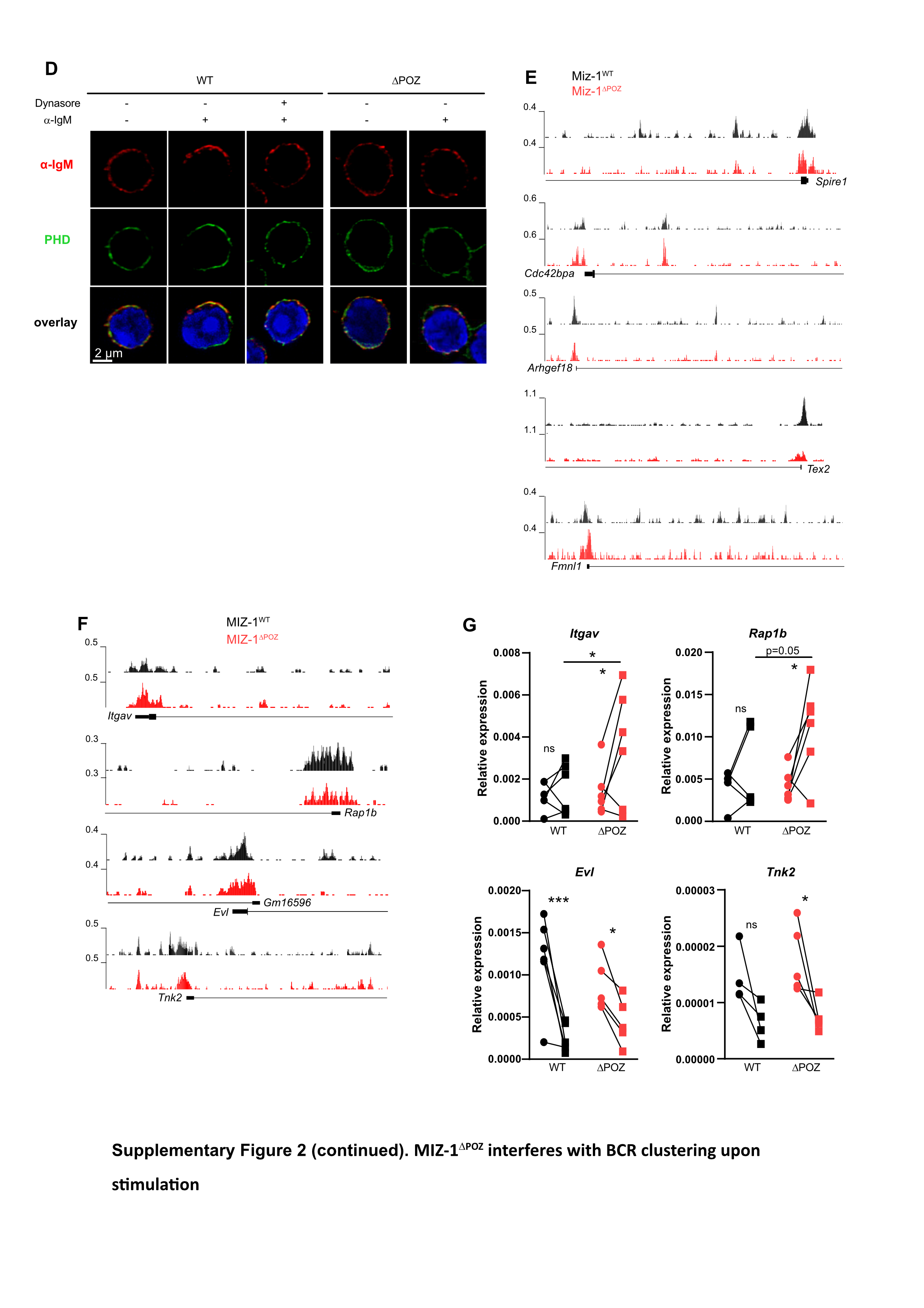

Supplement: Supplementary Figure 4 — MIZ-1ΔPOZ is associated with apoptosis of MYC-overexpressing B cells. (A-B) FACS analysis of splenic B cell populations from MIZ-1WT (WT), MIZ-1ΔPOZ (ΔPOZ), Eμ-Myc (MYC) and Eμ-Myc MIZ-1ΔPOZ (MYC ΔPOZ) mice. (A) percentage of CD19+ population relative to total splenocytes (B) percentage Annexin V+ cell from the CD19+ population. Ordinary one-way ANOVA with Tukey’s multiple comparisons test was used for statistical analysis (ns: not significant, *: p<0.05; **: p<0.01; ***: p<0.001; ****: p<0.0001). (C) Western blot showing MYC protein expression in B220+ splenocytes from MIZ-1WT (WT), MIZ-1ΔPOZ (ΔPOZ), Eμ-Myc (MYC) or Eμ-Myc MIZ-1ΔPOZ (MYC ΔPOZ) mice. Lamin was used as a loading control. [file Image4.tiff]

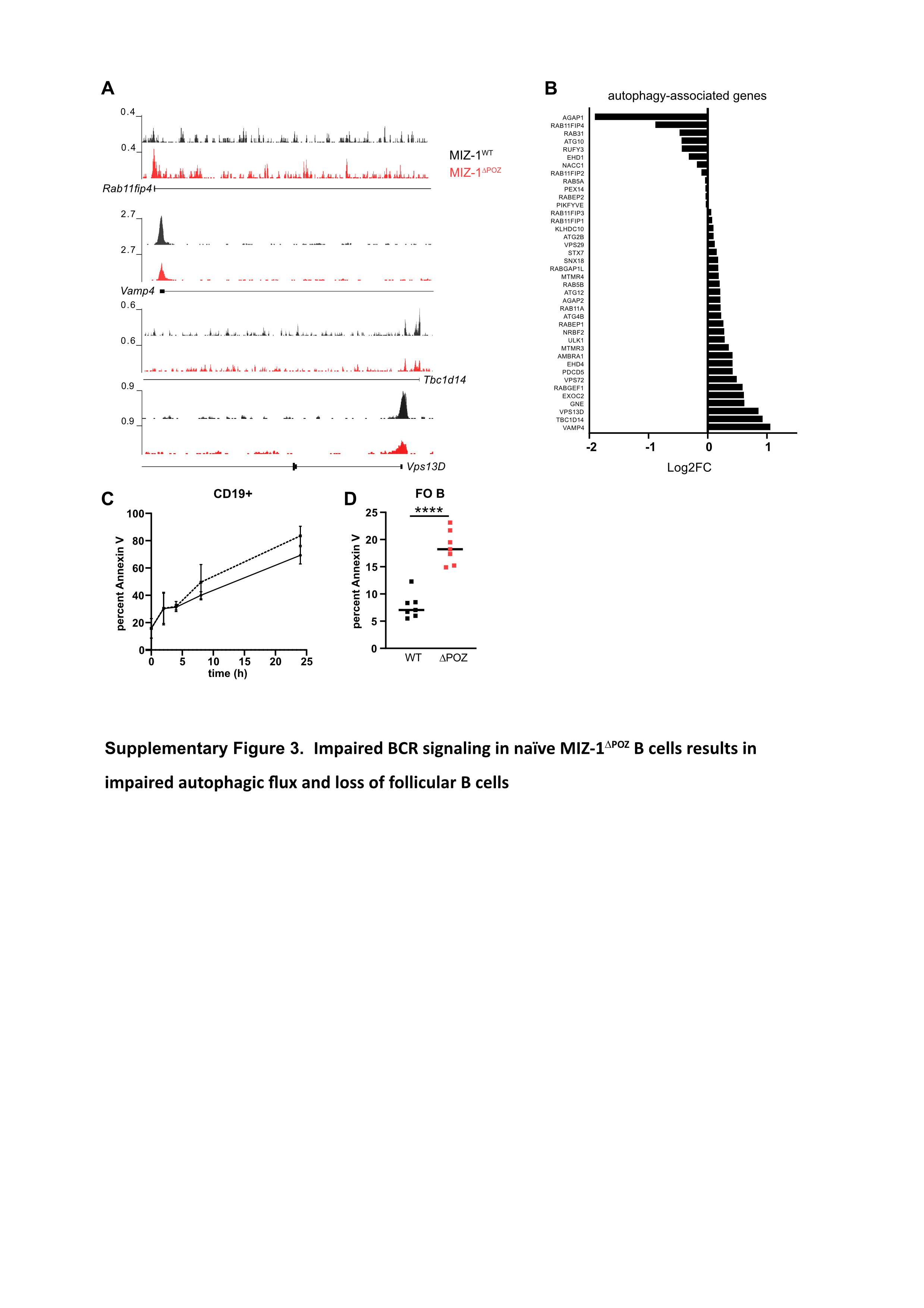

Supplement: Supplementary file 8 [file Image5.tiff]

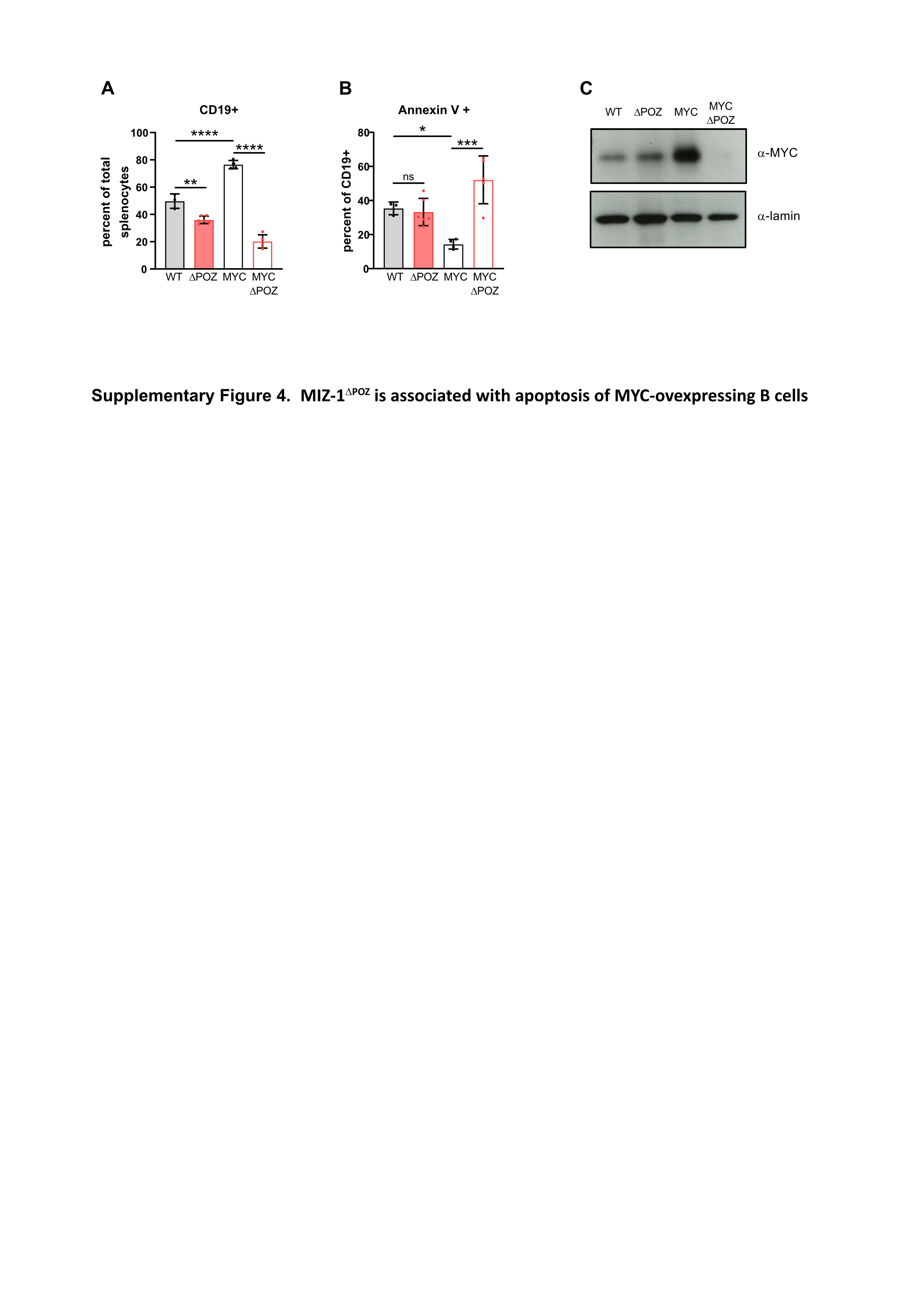

Supplement: Supplementary file 9 [file Image6.tiff]
